# Automated Individualization of Size-Varying and Touching Neurons in Macaque Cerebral Microscopic Images

**DOI:** 10.3389/fnana.2019.00098

**Published:** 2019-12-17

**Authors:** Zhenzhen You, Yaël Balbastre, Clément Bouvier, Anne-Sophie Hérard, Pauline Gipchtein, Philippe Hantraye, Caroline Jan, Nicolas Souedet, Thierry Delzescaux

**Affiliations:** ^1^CEA-CNRS-UMR 9199, Laboratoire des Maladies Neurodégénératives, MIRCen, Université Paris-Saclay, Fontenay-aux-Roses, France; ^2^School of Computer Science and Engineering, Xi’an University of Technology, Xi’an, China

**Keywords:** neuron individualization, touching neurons, size-varying neurons, microscopic images, macaque brain

## Abstract

In biomedical research, cell analysis is important to assess physiological and pathophysiological information. Virtual microscopy offers the unique possibility to study the compositions of tissues at a cellular scale. However, images acquired at such high spatial resolution are massive, contain complex information, and are therefore difficult to analyze automatically. In this article, we address the problem of individualization of size-varying and touching neurons in optical microscopy two-dimensional (2-D) images. Our approach is based on a series of processing steps that incorporate increasingly more information. (1) After a step of segmentation of neuron class using a Random Forest classifier, a novel min-max filter is used to enhance neurons’ centroids and boundaries, enabling the use of region growing process based on a contour-based model to drive it to neuron boundary and achieve individualization of touching neurons. (2) Taking into account size-varying neurons, an adaptive multiscale procedure aiming at individualizing touching neurons is proposed. This protocol was evaluated in 17 major anatomical regions from three NeuN-stained macaque brain sections presenting diverse and comprehensive neuron densities. Qualitative and quantitative analyses demonstrate that the proposed method provides satisfactory results in most regions (e.g., caudate, cortex, subiculum, and putamen) and outperforms a baseline Watershed algorithm. Neuron counts obtained with our method show high correlation with an adapted stereology technique performed by two experts (respectively, 0.983 and 0.975 for the two experts). Neuron diameters obtained with our method ranged between 2 and 28.6 μm, matching values reported in the literature. Further works will aim to evaluate the impact of staining and interindividual variability on our protocol.

## Introduction

The brain is the main part of the central nervous system and controls most body functions. It is constituted by a network of billions of neurons that range from 5 to 30 μm in diameter (Andersen et al., 2016). Information about the number, morphology (size and shape, etc.), and distribution (density and orientation, etc.) of neurons is essential to study brain development in health and disease. Such studies include development, aging (Williams and Herrup, 1988; Pakkenberg and Gundersen, 1997; Larsen et al., 2006; Pelvig et al., 2008; Karlsen and Pakkenberg, 2011; Walløe et al., 2014), cyto-architecture (Andrey et al., 2010; Andersen et al., 2016), and neurodegeneration (Thu et al., 2010; Waldvogel et al., 2015). However, these studies are challenging because of the varying size and color intensity of the neurons, their high density in certain regions, and the large information content of cellular-scale images. In this article, we define touching neurons as neurons that are in contact and/or neurons that seem to overlap because of the implicit projection of a three-dimensional (3-D) sample into a two-dimensional (2-D) image. In practice, stereology (Gundersen, 1986; West et al., 1991; Jelsing et al., 2006) is the reference method used by neurobiologists to estimate the number of cells in a region of interest (ROI). This technique is robust and unbiased when properly used but relies on long and tedious manual interventions. Moreover, the accuracy of the measurement depends mainly on three factors: (1) the complexity of the anatomical regions (cell density and organization), (2) the parameters of the method (such as the number or the size of sections to be considered and sampling of the optic dissectors that are necessary to be adjusted), and (3) the experience of the operator in stereology.

Today, more advanced techniques and more exhaustive studies on cell individualization are under development using new automated image processing methods. Conversely, to stereology, which takes into account the whole thickness of a sample and can deal with cells that are only partially embedded in the field of view, image processing methods only have access to flattened 2-D images. Mathematical morphology (Nedzved et al., 2000; Shu et al., 2013) can be applied to segment partially touching cells using the opening operation or the ultimate residues, but only strives in low-density regions. Approach based on concavity detection (Bai et al., 2008; Kothari et al., 2009; Zhang et al., 2013; Qi, 2014; Riccio et al., 2019) allows concave points on the contours of touching cells to be detected. Touching cells can be optimally separated using ellipse registration or a distance transformation algorithm, but false concave points due to noise are often present. It is not possible to apply this method to high-density cases where concavities may not be present. Region growing (Zucker, 1976; Adams and Bischof, 1994) can separate touching cells if appropriate seeds are detected. Otherwise, the non-detection or the overdetection of seeds will lead to, respectively, undersegmentation or oversegmentation. The Watershed algorithm is widely used to separate touching cells (Cousty et al., 2009) but easily generates oversegmentations and undersegmentations mainly due to noise in the images. To alleviate oversegmentation, algorithms have been proposed to select appropriate initial seeds (Yang et al., 2006; Lee et al., 2013; Shu et al., 2013; Dewan et al., 2014; Xu et al., 2014). Likewise, if appropriate initial contours are obtained, active contours can also avoid oversegmentation (Li et al., 2008). Graph-cut methods (Daněk et al., 2009; Al-Kofahi et al., 2010; Lou et al., 2012) are also popular today because they are robust to noise and integrate visual information and topological constraints. Nevertheless, these methods do not solve the problems of oversegmentation and undersegmentation even in simple cases where only a few cells are aggregated. The iCut algorithm (He et al., 2015) was proposed to individualize touching cells but fails to separate densely clustered cells in very dense regions [e.g., dentate gyrus (DG), a subregion of the hippocampus] due to the absence of concave points. In addition, this method produces non-natural regular individualization results because of the *a priori* fixed cell size. In recent years, the emergence of deep learning techniques has led to several applications for the analysis of complex cells of histology sections (Kainz et al., 2015; Zhang et al., 2015; Xie et al., 2016). In Kainz et al. (2015), a function of the distance to the center of the closest cell is designed to identify cell centers. However, a parameter corresponding to the average object size needs to be fixed *a priori* and cannot be adapted, making it poorly adapted to size-varying cells or very dense regions. A Fully Convolutional Regression Network (FCRN, Xie et al., 2016) was proposed to perform a regression of a cell spatial density map, providing an estimate of the number of cells. Nevertheless, the model considers a fixed model of Gaussian at the center of each cell (with *σ* = 2) that cannot be adapted to size-varying cells either. Furthermore, the authors reported that their method gives incorrect prediction in the case where a roughly rounded cell is clumped with four or more cells. Yet, regions like the DG contain thousands of aggregated cells. Deep learning, in addition, requires a large number of manually segmented training images and is computationally expensive. So far, a limited number of methods have been proposed for individualizing touching cells due to the complexity of the problem and the diversity of the configurations (cell type, immunohistochemistry staining, and digitization systems, etc.). In the case of a large number of aggregated neurons (e.g., DG), none of these methods can produce satisfactory results. Furthermore, most of the previous studies have been performed on specific data presenting stable object size or density that make these methods neither generic nor adapted to size-varying objects such as neurons.

This article reports a new image processing protocol aiming to automatically individualize size-varying and touching neurons and offers a rigorous and extensive validation. The experiment was performed on macaque brain sections stained by immunohistochemistry using the neuronal nuclei (NeuN) antibody. Noise in the digitized images was reduced by Gaussian filtering. Due to the large uncertainty about neuron sizes, this denoising step should be self-adaptive. Through an original enumeration approach, values of the Gaussian filter width were tested in a realistic range, and the optimal one was selected when locally stable individualization results were produced at the cellular level. Neuron center location and boundary information were enhanced by min-max filtering (You et al., 2016). Finally, neuron individualization was performed using a contour-based model. The individualization results obtained in this study are promising. The *F*-score of neurons counting using our approach is equal to 0.816 ± 0.062 in the ROIs and can achieve a higher score of 0.905 ± 0.001 in the subiculum. To the best of our knowledge, the proposed method presents the unique ability to process massive touching neurons in the DG with an *F*-score superior to 0.85. Moreover, the proposed method was ultimately compared with an adapted stereology technique, which is the gold standard technique used in biology. The obtained results show a high linear correlation with stereology (respectively, 0.983 and 0.975 for the two experts) which makes our approach promising for further biological studies.

Our contributions are as follows. (1) We proposed an original multiscale protocol to individualize size-varying and numerous touching neurons. (2) We built three reliable image datasets that are large, highly variable, and representative of the complexity of the macaque brain (anatomical regions, neuron density, neuron size) from 17 major anatomical regions. (3) We manually generated four large reference databases as ground truth with the contributions of five experts in the domain of biology and image processing (segmentation and counting). (4) The proposed method was compared with Watershed visually and quantitatively with several indexes. (5) Originally, the proposed method was compared with adapted stereology.

## Materials and Methods

### Biological Material

All animal studies were conducted according to French regulations (Directive 2010/63/EU–French Act Rural Code R 214-87 to 131). The animal facility is authorized by veterinarian inspectors (authorization no. D 92-032-02) and complies with Standards for Humane Care and Use of Laboratory Animals of the Office of Laboratory Animal Welfare (OLAW–no. #A5826-01).

**FIGURE 1:**
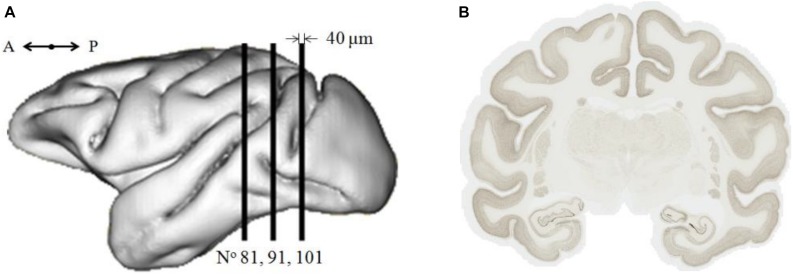
**(A)** Location along the rostro-caudal axis of the three selected coronal sections (sagittal view). A, anterior; P, posterior. **(B)** Image of brain section No. 91 of 230,000 pixels × 188,000 pixels (50.74 mm × 41.33 mm), ∼145 GB.

This work was performed on a 9-year-old healthy adult male cynomolgus macaque, *Macaca fascicularis*. After euthanasia, its brain was extracted from skull and frozen. The brain was then cut into 40-μm-thick serial coronal sections. Eight interleaved series were produced, leading to 0.32-mm interspace between two consecutive sections of a series. About 133 brain sections of one series were stained using a neuronal marker NeuN. These sections were digitized using a whole-slide imaging bright field scanner (Axio Scan.Z1, Zeiss) with an in-plane resolution of 0.22 μm (×20 magnification). In the current work, a subset of three brain sections, the 81st, the 91st, and the 101st section (noted Nos. 81, 91, and 101; Figure 1), along the rostro-caudal axis from the frontal pole to the caudal end of the cerebral cortex were selected and processed. They are representative of the most frequent neuron distributions found in the entire brain. They contain 17 major anatomical regions (Supplementary Table 1): cortex, hippocampus, thalamus, and so on. Each digitized brain section corresponds to about 140 GB of data.

### Datasets

As the staining protocol was performed simultaneously for all analyzed histological sections, the staining is assumed to be similar among the sections treated, without bias in staining intensity. Sections Nos. 81, 101 were selected for learning segmentation of neuronal staining and section No. 91 for testing the individualization method. Then, three different datasets were produced for this study.

To learn how to segment neuronal staining, a segmentation dataset of a hundred representative images (512 pixels × 512 pixels) from sections Nos. 81, 101 were extracted (Supplementary Figures 1–3 and Supplementary Table 2) and manually segmented into three classes (neuronal staining, non-stained tissue, and background) by an expert.

To validate the individualization method, an individualization dataset of 58 images (5,000 pixels × 5,000 pixels) extracted from section No. 91 (Supplementary Figures 4, 5 and Supplementary Table 3) presenting different neuron densities and different anatomical regions was created. In these images, each neuron was manually identified by an expert by a point in its center, noted centroid whose intensity is the darkest among its neighbors, providing information about the location and the number of neurons (about 0–4,000 neurons per image).

To compare our method with stereological neuron counting, an anatomical stereology dataset including eight large images (including from 3 × 10^6^ to 1.2 × 10^8^ pixels) selected from six anatomical regions presenting a wide range of neuron densities were generated (Supplementary Figure 6). Neuron counting methods based on 2-D image processing deal with flattened images (single focus setting), which do not allow us to differentiate entire neurons present in the histological section from partial neurons produced at the surface of the section during the cutting process. We chose the optical dissector method as a reference to evaluate our methodology, but we did not take into account dead zones to be able to compare the two approaches. In this way, it was possible to evaluate the individualization process, but a small bias was introduced in the counting process, possibly resulting in overdetection due to partially embedded neurons. Two biologist experts estimated the number of neurons on these images directly under a microscope (Leica DM6000) with the software Mercator (Explora Nova, Supplementary Figure 7) using an adapted stereology technique.

**FIGURE 2:**
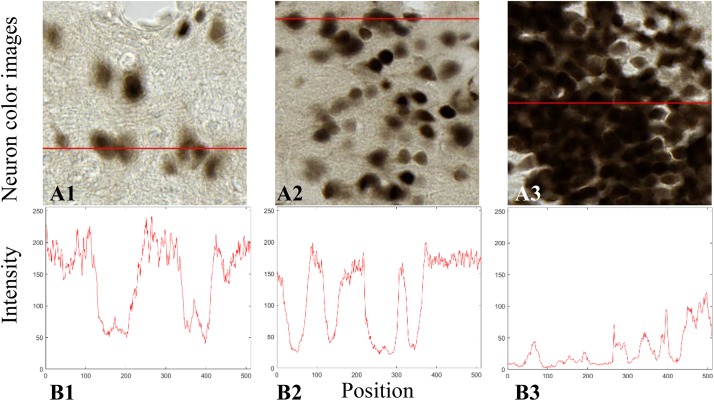
Transverse profiles of pixel intensity **(B1–B3)** in three histological images from anatomical regions of thalamus **(A1)**, cortex **(A2)**, and dentate gyrus (DG) **(A3)**.

The histological sections were stained by NeuN whose expression is present in the nucleus as well as in the cytoplasm of the neurons. The staining is darker in the nucleus and lighter in the cytoplasm. Figure 2 shows the transverse profiles of pixel intensity in three histological images ranging from low to high neuron densities. Neurons stained by NeuN are considered to be compact in this study (disk shape approximation). The reverse intensity of the transverse profile was considered to be similar to a Gaussian distribution. Therefore, we modeled this spatial profile by a Gaussian distribution with *σ_*n*_* as parameter, which can be seen as the probability density of a pixel being a neuron pixel. *σ_*n*_* can be evaluated by the diameter *d* of a neuron of interest (NOI) according to the three-sigma rule (Pukelsheim, 1994):

(1) $$\begin{array}{c} P_{r}\left(\mu -3\sigma_{n}\leq x\leq \mu +3\sigma_{n}\right)\approx 0.9773\\ 2\times 3\,\sigma_{n}+1\leq d \end{array}$$

where *μ* is the expectation, interpreted as neuron centroid, and *σ_*n*_* is the spatial standard deviation, related to the neuron size. *x* is an observation from Gaussian distributed random variable.

Considering that the diameter of the largest neuron is 30 μm (Andersen et al., 2016) (about 136 pixels at × 20 magnification), we calculated that the value of *σ_*n*_* is inferior to 23 pixels according to the spatial resolution used in this work. Taking into account the appropriate sampling for computation test, we investigated integer values of *σ_*n*_* ranging from 1 to 23 pixels.

**FIGURE 3:**
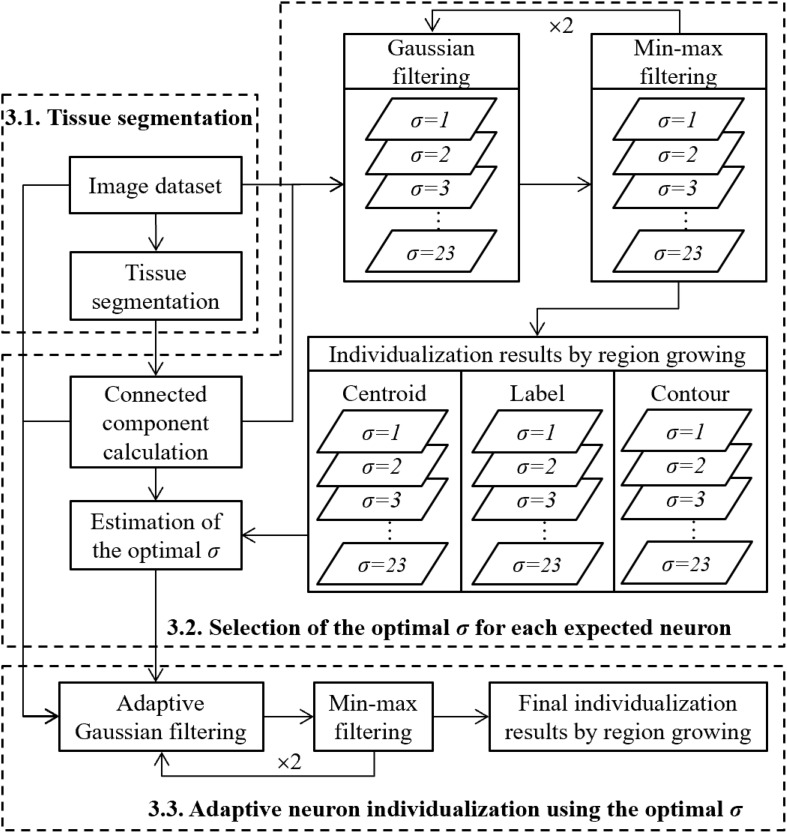
Global strategy to perform the individualization of neurons.

To segment individual neurons, the general idea was to first extract pixels corresponding to the neuron class and then separate touching neurons, which can be transformed into a problem of detection of the centroids of the neurons. Prior to the detection, a previous denoising step was performed by applying Gaussian filter parameterized by *σ*. Local minima corresponding to expected centroids can then be detected by the proposed min-max filter, which is able to enhance in a robust way the information of the centroids as well as the contours of neurons (see section “Centroid Detection Based on Min-Max Filter”). The choice of the parameter *σ* influences the final detection result. A small value of *σ* cannot remove all of the noise leading to the overdetection of the centroids by min-max filter, whereas a large value of *σ* will over smooth the image leading to the underdetection of the centroids. We supposed in this paper that the centroids can be correctly detected when the parameter of the Gaussian filter is adapted to that of Gaussian model of the NOI considered. Thus, the parameter *σ* of the Gaussian filter was defined by the *σ_*n*_* estimated on the NOI. In the case of touching neurons, the estimation of *σ_*n*_* was calculated by analyzing all of the individualization results, associated to all possible values of *σ* (ranging from 1 to 23 pixels). We assumed that *σ_*n*_* was the one associated with the local stable individualization result. In practice, this was obtained by considering at least two consecutive *σ*, which gave stable individualization results (see section “Estimation of the Optimal *σ*”). Once the optimal *σ* to be used for each neuron was estimated, we applied the adaptive Gaussian filter to reduce noise to detect centroids of neurons of different sizes, and we performed the final individualization. The general workflow of the proposed methodology is presented in Figure 3.

### Tissue Segmentation

A Random Forest (RF) model (Breiman, 2001), including 100 decision trees for tissue segmentation, was generated using the *segmentation dataset*, which was divided into two subsets, a learning set and a validation set. Seventeen features (R, G, B, H, S, V, X, Y, Z, L^∗^, a^∗^, b^∗^, M, V, LBP10, LBP40, and LBP63) were studied to produce the RF model. Thereinto, four main color spaces defined by CIE (Commission Internal de l’Eclairage) were considered. They are RGB (Red, Green, and Blue), HSV (Hue, Saturation, Value), XYZ (one CIE color space, which represents perceptual uniformity), and L^∗^a^∗^b^∗^ (a typical CIE color space transformed non-linearly from XYZ) (Cheng et al., 2001). M and V are, respectively, the mean value and variance of gray-level intensity computed in a cross of 10 pixels. Local binary pattern (LBP) is a texture feature (Wang and He, 1990; Ojala et al., 2002) computed in a disk. The radius of this disk is fixed to 10, 40, and 63 pixels in this study, which correspond to the radiuses of small, average, and large neurons. To produce the RF model with optimal features while keeping satisfactory segmentation performance, we selected four features, L^∗^, M, V, and LBP40, which were progressively selected while keeping the best candidate based on successive tests performed from the entire set of features. This selection of features was confirmed in previous work, aiming to objectively determine optimal features and which pointed out that numerous combinations were able to produce proposer segmentation (Vandenberghe et al., 2015; Bouvier et al., 2018). This strategy of selection provided relevant, limited, and intelligible features compared to deep learning techniques, which can be assimilated to black boxes. Previous works performed on histological sections supported our choice as well based on the robustness of this approach (Vandenberghe et al., 2015, 2018).

The *individualization dataset* was then segmented into three classes based on the RF model produced. In practice, a median filter (7 pixels × 7 pixels) was applied to reduce impulsional noise and to regularize neuron contours in binary images. Then, connected components presenting an area inferior to a third of the surface of the smallest neuron (estimated to 127 pixels) were removed. This step can possibly remove partial neurons detected, as their size is insufficient to be counted. The binary image of neuron class produced was noted by *I*_*m*_.

### Selection of the Optimal *σ* for Each Expected Neuron

The following procedure was performed on each connected component, which was calculated based on *I*_*m*_.

As neuron size is variable in the brain (ranging from 5 to 30 μm in diameter), it is important to estimate the optimal *σ* of the Gaussian filter to be applied to properly detect the centroids of size-varying neurons in multiple brain regions using min-max filter. The parameter of the optimal *σ* of Gaussian filter was estimated in the following three steps.

#### Centroid Detection Based on Min-Max Filter

The detection of neuron centroids was performed on the grayscale image *I*_*g*_, which is the average of R, G, and B channels of the original color image (Hanbury, 2008). The grayscale image was filtered with a Gaussian kernel (*I*_*gf*_), and non-neuron pixels were masked out (*I*_*gfm*_). This image was used as an input to an original min-max filter (You et al., 2016) resulting in an extrema map (*I*_*e*_). Briefly, for each pixel *o* of *I*_*gfm*_, let *D(r, o)* be the disk (not including *o*) of radius *r* centered on *o*, *N(D)* the number of pixels in *D(r, o)*, *Max(o)* the number of pixels whose intensity value in *D(r, o)* is inferior or equal to *I_*gfm*_(o)* and *Min(o)* the number of pixels whose intensity value is superior to *I_*gfm*_(o)*. The calculated value was then:

(2) $$I_{e}\,\left(o\right)=\frac{\mathrm{Max}\,\left(o\right)-\mathrm{Min}\,\left(o\right)}{N\,\left(D\right)}$$

Consequently, pixels whose value is −1 are defined as minima in the local disk while those whose value is 1 are defined as maxima. *r* was fixed to 10 pixels corresponding to the expected minimum neuron radius (2.5 μm). The two-step process (Gaussian filtering combined with min-max filtering) can be repeated iteratively *n* times to refine the extrema map. For densely clustered neurons, performing this process one time is not sufficient to distinguish them since their staining intensities are too similar or even equal to extract ideal minima. In addition, in a few cases, if the intensity of one neuron is entirely higher than that of its neighboring neurons, its corresponding minima could not be determined. On the contrary, multiple iterations would lead to excessive enhancement since equation (2) is iteratively computed in the disk of fixed radius *r*. Therefore, a compromise needs to be found. *n*, the number of iterations, was set to *n* = 2 (see section “Number of Iterations–*n”*). For a given value of *σ*, pixels of value −1 were selected as neuron centroids, and each centroid was assigned a unique label (*id*).

#### Neuron Individualization by Competitive Region Growing

Neuron individualization was performed based on the use of a discrete contour-based model. Contours were initialized as *r*_0_-pixel-radius (1<*r*_0_<10) circles centered on each centroid, and all pixels inside the contours were assigned with their centroids’ label (*id*). As the intensities of neuron boundaries in *I*_*e*_ are close to its maxima, we proposed to give each contour point an expanding speed that depends on the contour curvature and *I*_*e*_’s intensity. If the contour curvature on a point is smaller and/or the *I*_*e*_’s intensity on a point is darker (distant from the boundary), the expansion speed should be faster, and vice versa. Let *p* be a contour point, *κ(p)* the curvature-dependent term, *h(p)* the intensity-dependent term, and *o* the position of the neuron centroid. The next position *p’* of *p* was calculated as:

(3) $$\vec{{\mathrm{op}}^{{}^{\prime }}}=\vec{\mathrm{op}}+\frac{\vec{\mathrm{op}}}{\mathrm{op}}\times \kappa \,\left(p\right)\times h\,\left(p\right)$$

Let *k(p)* be the contour curvature at point *p* and *c* a predefined curvature chosen by the user. **K*(p)* was calculated by equation (1), making the contour expanding for curvatures smaller than *c* (*k(p) < c*) and shrinking for curvatures greater than *c* (*k(p) > c*), which was calculated as:

(4) κ⁢(p)=c-k⁢(p)

The intensity-dependent term was inspired by the work of Perona and Malik (1990). Let *t* be a coefficient empirically set to 0.8 × max(*I*_*e*_). *h(p)* was calculated as:

(5) $$h\,\left(p\right)=\exp\,\left(-{\left(\frac{I_{e}\,\left(p\right)+1}{2\,t}\right)}^{2}\right)$$

After each progression, the contour was smoothed by a mean filter. It was implemented for each contour point by taking the average position information of its two adjacent neighbor points. Then, the distance between two consecutive points *p* and *q* was examined; if it was superior to a predefined maximal distance *d*_*max*_, new points were interpolated automatically according to:

(6) $$\left\{ \begin{array}{c} N=\frac{\vec{\mathrm{pq}}}{d_{\max}}-1\\ \vec{{\mathrm{op}}_{i}}=\vec{\mathrm{op}}+\frac{\vec{\mathrm{pq}}}{N+1}\times i \end{array}\right.\;1\leq i\leq N$$

where *N* represents the number of new points to interpolate, and *p*_*i*_ is the *i*th point to interpolate.

Pixels around each contour point *p* within *d*_*max*_ distance were examined. Those pixels nearer to their centroid compared to *p* and not yet labeled were assigned their centroid’s *id*.

All cell contours in a connected component produced during neuron class segmentation were simultaneously expanded, and contour crossing was forbidden. The possibility to perform individualization on connected components makes it possible to massively parallelize the process. In our experiment, we fixed the number of expanding iterations at 100. This number can be arbitrarily high and should be chosen so that the computation time is low enough to enable the contours to reach every neuron boundary. When several cell contours were expanded too close, the new expansion location may jump into another cell. It was forbidden, and the expansion stopped. At the end of this process, unlabeled pixels may exist, and they were assigned to a label according to their neighbors in a 3 × 3 window by majority voting.

#### Estimation of the Optimal *σ*

This individualization method using a single *σ*-fixed parameter for the entire dataset has resulted in overindividualization and under individualization due to inappropriate value of *σ* to treat neurons presenting different sizes. A multiscale strategy was thus integrated in this work to deal with this major issue. We then tested all possible values of *σ* to obtain information related to centroids, labels, and contours of neurons, and we estimated the optimal *σ* for every expected neuron inside each connected component. We analyzed the relationship between *σ*-dependent contour image and the map of accumulated contours calculated by summing all *σ*-dependent contours to estimate the optimal value of *σ* for an expected neuron. Our hypothesis was that consistent consecutive values of *σ* should produce similar or close contours that will be accumulated in the same location.

**FIGURE 4:**
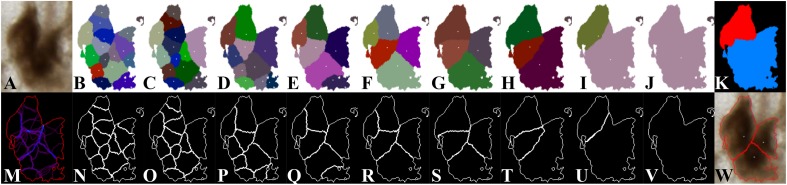
*σ*-dependent individualization results. **(A)** Original image of a connected component including three neurons. **(B–H)** Individualization results for *σ* ranging from 1 to 7. **(I)** Close individualization results for *σ* ranging from 8 to 11. **(J)** Individualization result for *σ* ranging from 12 to 23. **(N–V)** Contours of the individual neurons corresponding to **(B–J)**. The different colors in **(B–J)** represent the individual neurons, and the white points represent the detected centroids. The white curves in **(N–V)** represent the contours of the individual neurons. **(M)** Map of accumulated contours, summation of all *σ*-dependent contours. Violet color represents the minimum value (1 contour); blue-like color represents overlapped contours, which are candidate for individualization of touching neurons; and red color represents the maximum value (23 cumulated contours). **(K)** Map of the optimal *σ* determined. The color blue represents *σ* = 5, and the red represents *σ* = 12. **(W)** Illustration of the individualization results produced by the proposed method. The white points represent the detected centroids, and the red curves are the final neuron contours.

Figure 4 shows the individualization results depending on *σ* values. Figure 4A presents the original image of a connected component including three neurons. Figures 4B–J display the *σ*-dependent label images overlapped with detected centroids. Figures 4N–V display the *σ*-dependent contour images. Figure 4M is the map of accumulated contours, and Figure 4K is the map of the optimal *σ* determined. Figure 4W illustrates the individualization result produced by the proposed method. In Figure 4I, close individualization results corresponding to the same number of neurons detected were obtained for consecutive values of *σ* ranging from 8 to 11. It was defined as a stable state (*E*). Figure 4J presents another stable state for *σ* ranging from 12 to 23. In this example, the stable states present when *σ* varies from 8 to 11 (*E*_1_, Figure 4I) and from 12 to 23 (*E*_2_, Figure 4J).

Next, we analyzed the *σ*-dependent neuron contours. The idea was to first synthesize this information on a map of accumulated contours (Figure 4M), which was calculated by summing *σ*-dependent contour for all possible values of *σ*. Points of higher intensity value in the map describe zones of higher probability to be contours between touching neurons. Because a connected component consists of at least one or multiple neurons and no prior information about neuron size is known, intensity values corresponding to real contours in the map may be different. Therefore, to find the optimal contours, we proposed to study all of the intensity levels that can be encountered in the map of accumulated contours using a threshold *s* ranging from 1 to 23. This approach enabled us to detect both small and high number of consecutive values of *σ* producing stable contours of neurons. A similarity criterion between the thresholded maps and each *σ*-dependent contour result was then calculated according to the Dice score:

(7) $$\mathrm{Dice}\,\left(\sigma ,s\right)=\frac{2\times N_{c}\,\left(\sigma ,s\right)}{N_{i}\,\left(\sigma \right)+N_{\mathrm{as}}\,\left(s\right)}$$

where *N*_*i*_ is the number of pixels in the *σ*-dependent contour result, *N*_*as*_ is the number of pixels in *s*-dependent thresholded map, and *N*_*c*_ is the number of pixels that exist in the same spatial location in both *σ*-dependent contour result and *s*-dependent thresholded map. This computation was performed at the connected component level. The Dice score varied between 0 and 1 (perfect superimposition).

Table 1 lists Dice scores computed for the connected component given in the example of Figure 4. The closer the value is to 1, the more the *σ*-dependent contour is similar to the *s*-dependent thresholded map. We then fixed a threshold of Dice value (*S*_*dice*_), which defines a sufficient similarity between two contours. This threshold is calculated as follows:

**TABLE 1:** Table of Dice scores computed in the example of Figure 4.

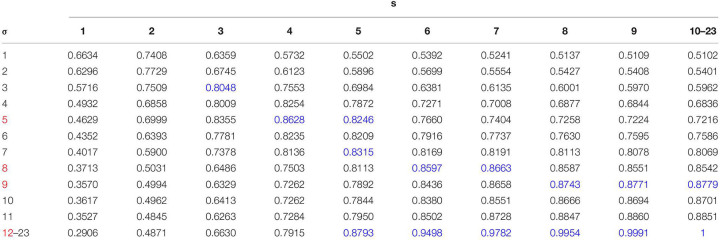

*The Dice scores vary between 0 and 1. The closer the Dice score is to 1, the more the *σ*-dependent contour is similar to the *s*-dependent thresholded map. Numbers in blue are superior to *S*_*dice*_ and are local maxima for each fixed value of *s*. It represents the corresponding *σ*, which provides locally the best individualization result. The detection of consecutive maximal Dice scores for a fixed *σ* corresponds to a locally stable segmentation, and this *σ* is a candidate (red color) for a subpart in this connected component (neuron).*

(8) $$\left\{ \begin{array}{c} S_{\mathrm{dice}}=\min_{E_{i}\in E}\left\{\sum_{\sigma \in E_{i}}\sum_{s=1}^{23}\mathrm{Dice}\left(\sigma ,s\right)/\left(23\times \mathrm{Card}\left(\sigma \in E_{i}\right)\right)\right\}\\ S_{\mathrm{dice}}=0.8\;i\,f\,S_{\mathrm{dice}}>0.8 \end{array}\right.$$

where *E*_*i*_ represents a stable state of the set *E* = {*E*_1_, *E*_2_, …, *E*_*m*_} (*m* ≤ 11, theoretically up to 11 stable states). *Card*(*σ ∈ E_*i*_*) is the number of *σ* belonging to the state of *E*_*i*_. 0.8 is a threshold parameter defined empirically.

In Table 1, given a fixed *s*, the blue numbers are superior to *S*_*dice*_ and are local maxima. It means that the corresponding *σ* provides locally the best individualization result. Given a fixed *σ*, when *s* increases, the effect of overindividualization is reduced, but conversely the risk of under individualization increases. If at least two consecutive maximal values of Dice exist, this *σ* is selected as a candidate. In the example of Table 1, candidates for the optimal *σ* are 5, 8, 9, and 12 (red color in *σ* column).

Then we generated the map of the optimal *σ* to save the values of the optimal *σ* to be applied for each neuron when applying adaptive Gaussian filtering. The pixels corresponding to neurons in this map were initialized to *σ* = 23. The study of the optimal *σ* was performed from the largest value of *σ* candidate to the smallest. For each *σ* studied, individualization results were compared with *σ*+1 result because *σ*−1 result probably leads to overindividualization. If any of the two NOIs individualized by *σ* and *σ*+1 were sufficiently similar [*Dice[NOI_*a*_(σ), NOI_*b*_(σ+1)]* > 0.95, neuron region reproducibility, equation (9)], this value of *σ* was selected as the optimal one for the individualized NOI and was updated in the map of the optimal *σ*. If not, *σ* kept its previous value.

$$\mathrm{Dice}\,\left({\mathrm{NOI}}_{a}\,\left(\sigma \right),{\mathrm{NOI}}_{b}\,\left(\sigma +1\right)\right)$$

(9) $$\;=\frac{2\times S\,\left({\mathrm{NOI}}_{a}\,\left(\sigma \right),{\mathrm{NOI}}_{b}\,\left(\sigma +1\right)\right)}{S\,\left({\mathrm{NOI}}_{a}\,\left(\sigma \right)\right)+S\,\left({\mathrm{NOI}}_{b}\,\left(\sigma +1\right)\right)}$$

where *NOI_*a*_(σ)* and *NOI_*b*_(σ+1)* represent, respectively, one NOI individualized by *σ* and *σ*+1, *S[NOI_*a*_(σ)]* and *S[NOI_*b*_(σ+1)]* represent, respectively, the surface of *NOI*_*a*_ individualized by *σ* and the surface of *NOI*_*b*_ individualized by *σ*+1, and *S[NOI_*a*_(σ), NOI_*b*_(σ+1)]* is the number of pixels that exist in the same spatial location in both two NOIs individualized. 0.95 was defined empirically.

Note that neuron density in the DG is the highest in the brain. As thousands of neurons are aggregated, the DG forms an extremely large connected component. Moreover, the stained neurons are similar to each other according to size in this region. The detection of the neurons in DG region is therefore very sensitive to the parameter *σ* of the Gaussian filter. However, neuron sizes in the DG have a low variability, making it possible to apply a fixed value of *σ*. To evaluate this *σ*, we selected all of the images including the DG from the individualization dataset (seven out of 58 images). Then, new binary images were generated by keeping only the DG. With these binary images, we computed the 23 *σ*-dependent individualization results using the proposed method and the ground truth (manual counting in the same regions). Using the evaluation method described in section “Comparison of Automated and Manual Neuron Counting,” the optimal *σ* was determined, and details will be provided in section “Optimal *σ* for the Region of the DG.” The DG represents a very small fraction of the total amount of data processed and was the only region requiring the general protocol to be adapted.

### Adaptive Neuron Individualization Using the Optimal *σ*

At this stage, we have defined an unsupervised strategy to automatically determine the optimal *σ* to be applied to each NOI. We then applied the two-step process (adaptive Gaussian filtering using the map of the optimal *σ* as parameter and min-max filtering) to detect the final centroids of neurons of different sizes. The two-step process was applied twice (optimized in section “Number of Iterations–*n”*) because according to the experimental results on real data, one iteration led to underdetection of centroids, and more than two iterations led to overdetection. The detected centroids were selected as initial seeds for region growing process based on the discrete contour-based model leading to the final individualization result.

### Evaluation Methods of Neuron Individualization

The proposed method was compared with the Watershed algorithm (Cousty et al., 2009), which defines the watershed cuts based on the principle of the drop of water on a topographic surface. As Watershed is sensitive to noise, a preprocessing step is necessary. We used Gaussian filter to reduce noise in the images from the individualization dataset. The choice of *σ* is crucial because we have no *a priori* information about neuron size. Therefore, Gaussian filter with the optimal *σ* computed for each NOI by the proposed method was applied before using Watershed algorithm.

The quality of neuron individualization was evaluated by three evaluation methods, which are introduced in the following parts.

#### Comparison of Automated and Manual Neuron Counting

The relative count error *ε* was used to validate the neuron count (Kothari et al., 2009). It was defined as the absolute difference between the automated (*N*_*a*_) and expert count (*N*_*e*_) divided by the expert count:

(10) $$\varepsilon =\frac{\left|N_{a}-N_{e}\right|}{N_{e}}$$

The smaller the relative count error is, the more accurate the automated method count.

In addition, a complementary individualization score that considers the colocalization of the neurons individualized using the automated method compared to the centroids pointed by the expert was defined. If exactly one expert centroid is superimposed on the neuron area automatically detected, this one is considered as a true positive. Otherwise, it is either oversegmented (zeros expert centroid) or undersegmented (more than 1 expert centroid). Recall (R), Precision (P), and *F*-score (F) were calculated according to:

(11) $$R=\frac{N_{t}}{N_{e}};P=\frac{N_{t}}{N_{a}};F=2\,\frac{R\times P}{R+P}$$

where *N*_*t*_ is the number of correctly automatically segmented neurons (true positive), *N*_*e*_ is the expert count, and *N*_*a*_ is the automatic counting of neurons.

The bigger the value of the *F*-score is, the better performance the method reaches. This validation method was widely used in previously published works (Al-Kofahi et al., 2010; Shu et al., 2013; Zhang et al., 2013; He et al., 2015; Poulain et al., 2015; Molnar et al., 2016).

These two validation methods are generic but do not take into account certain properties of the segmented neurons (e.g., individualization quality and shape/contour accuracy). This led us to propose a second method.

#### Study of the Location of the Centroids and the Contours of the Neurons

The interest of this approach is to consider local spatial information at the individual neuron level. The expert manually segmented individual neurons by marking their centroids and drawing their contours. Because this is a tremendous work that is time-consuming, we proposed a trade-off approach to produce preliminary results based on certain representative images and to assess the quality using the following indexes: the distances of the contours (*Distance_mean_contour*) or the centroids (*Distance_centroid*) between automated and manual individualization results, the overlapping fraction between neurons automatically and manually individualized (*Dice*_*area*_) [equation (12)]. Based on the individualization dataset, a representative subset of nine images corresponding to seven anatomical regions (caudate, claustrum, cortex, hippocampus, putamen, subiculum, and thalamus) was chosen. On each image, 100 neurons were randomly selected and segmented by an expert.

(12) $$\left\{ \begin{array}{c} d_{\min}\,\left(i,j\right)=\min_{j\in C_{\mathrm{auto}}}\left\{\sqrt{{\left(i_{x}-j_{x}\right)}^{2}+{\left(i_{y}-j_{y}\right)}^{2}}\right\}\\ d_{\mathrm{mean}}=\frac{1}{N_{\mathrm{manual}}}\,\sum_{\begin{array}{c} \#\,i\in C_{\mathrm{manual}}\\ \#\,j\in C_{\mathrm{auto}} \end{array}}d_{\min}\,\left(i,j\right)\\ {\mathrm{Dice}}_{\mathrm{area}}=\frac{2\times S_{\mathrm{common}}}{S_{\mathrm{manual}}+S_{\mathrm{auto}}} \end{array}\right.$$

where *N*_*manual*_ is the number of points in the contour drawn by the expert, *d*_*min*_ is for each point *i(i_*x*_,i_*y*_)* of the contour drawn by the expert, the minimal distance with the point *j(j_*x*_,j_*y*_)* in the contour delineated by the automatic method, *d*_*mean*_ is the average distance between neuron contours segmented manually and automatically, *C* represents neuron contour, *S*_*manual*_ is the neuron surface derived from the contour drawn by the expert, *S*_*auto*_ is the neuron surface segmented by the automatic method, and *S*_*common*_ is the overlapped area between manual and automatically segmented neurons.

These metrics allow different automated individualization methods to be efficiently evaluated. However, this work was limited due to the important number of manual operations required. *A posteriori*, the methods listed above (section “Comparison of Automated and Manual Neuron Counting” and section “Study of the Location of the Centroids and the Contours of the Neurons”), respectively, implied to manually deal with about 112,000 neurons in 50 images (5,000 pixels × 5,000 pixels) and 900 neurons in nine images in the individualization dataset.

The gold standard method for estimating the number of cells in an anatomical region is stereology. This led us to the third evaluation method.

#### Comparison of Automated Neuron Counting Versus Stereology

Biologists were asked to estimate the number of neurons in the stereology dataset (eight regions) with quantitative stereological counting techniques based on optical dissectors. Compared to classical stereological approach, dead zone regions were not considered to make possible to compare the results with our counting methodology. Two experts counted the neurons in samples from every anatomical region directly under a microscope using the software Mercator. Then, the number of neurons was estimated according to the optimal sampling fraction (two settings tested), and the two counting results were compared (method vs. experts).

## Results

Before presenting the results, it is important to mention that the individualization dataset consists of 58 images, in which eight images were discarded. They concern four blurred images (one image of caudate, two images of cortex, and one image of putamen) for which the expert cannot give ground truth and four images without staining tissue (two images of white matter and two images of ventricle).

### Parameters Optimization

#### Number of Iterations–*n*

**FIGURE 5:**
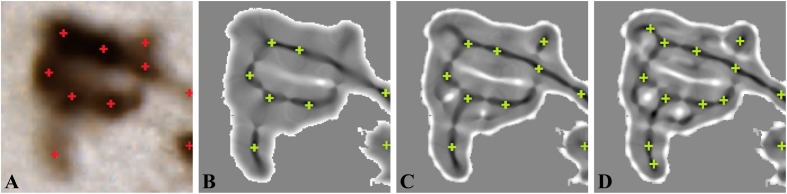
Min-max map computation (*σ* = 4). **(A)** Original color image. **(B)** One iteration *I*_*e*_. **(C)** Two iterations *I*_*e*_. **(D)** Three iterations *I*_*e*_. Red crosses are neuron centroids marked by the expert, whereas green crosses are the detected neuron centroids figure from You et al. (2016).

Example of neuron detection with different number of iterations is shown in Figure 5. We found that a consistent estimation between the expert and automated method was obtained for two iterations (10 centroids detected) because one iteration of min-max map computation leads to underdetection of neurons and three iterations to overdetection. The setting of two iterations of min-max map computation enabled to detect small, blurred, as well as bright neurons.

**FIGURE 6:**
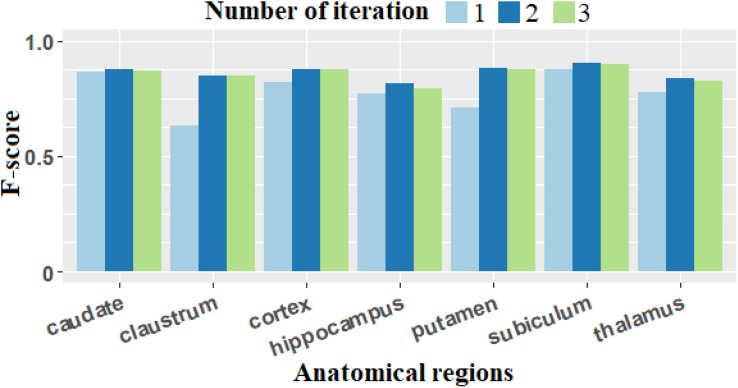
*F*-score computed with different number of iterations. The *F*-score presents the biggest values for all anatomical regions when we performed two iterations of the two-step process (adaptive Gaussian filtering and min-max filtering).

To generalize this illustrative example, we extended this study to the individualization dataset. The relative count error *ε* (Table 2) and the *F*-score (Figure 6) were computed by grouping the images corresponding to the same anatomical regions (50 images grouped into seven groups: caudate, claustrum, cortex, hippocampus, putamen, subiculum, and thalamus).

**TABLE 2:** Relative count error based on the number of iteration.

**Anatomical regions**	**Number of iteration – *n***		
	**1**	**2**	**3**
Caudate	0.163 ± 0.057	**0.073 ± 0.073**	0.111 ± 0.067
Claustrum	0.520 ± 0.051	0.074 ± 0.003	**0.028 ± 0.015**
Cortex	0.248 ± 0.055	0.051 ± 0.033	**0.048 ± 0.045**
Hippocampus	0.231 ± 0.179	**0.186 ± 0.161**	0.276 ± 0.221
Putamen	0.427 ± 0.183	0.085 ± 0.081	**0.066 ± 0.086**
Subiculum	0.185 ± 0.023	**0.033 ± 0.026**	0.047 ± 0.028
Thalamus	0.302 ± 0.050	**0.167 ± 0.061**	0.217 ± 0.063
Average	0.257 ± 0.131	**0.105 ± 0.114**	0.137 ± 0.167

*The values in bold are the smallest average value of relative count error among three iterations for each anatomical region.*

From Table 2, we observe that for all of the anatomical regions, the smallest average value of the relative count error (0.105 ± 0.114) is obtained for two iterations. Looking at the results region by region, we find that in the cortex, the relative error is similar for *n* = 2 and *n* = 3, but the standard deviation is smaller for *n* = 2, which provides more stable individualization results. In the claustrum and putamen, although the relative count error is smaller for *n* = 3, the standard deviation is greater. Considering the average relative count error, *n* = 2 is consequently the optimal processing to perform.

For *F*-score based on Figure 6, the value for *n* = 2 provides the best scores for all anatomical regions.

In summary, the optimal number of iterations selected is 2.

#### Optimal *σ* for the Region of the DG

It was mentioned previously that the DG is a particular case. *σ* = 5 is the optimal value of *σ* determined using *F*-score criteria [equation (11)].

**FIGURE 7:**
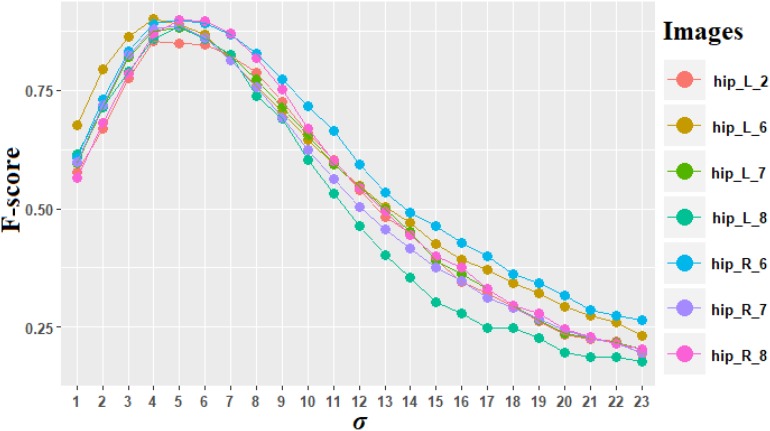
*F*-score computed for different values of σ for the region of dentate gyrus (DG). The different colors represent the seven images of DG. “hip” is the abbreviation for the name of the anatomical region of the hippocampus.

Figure 7 shows the *F*-score for *σ* varying from 1 to 23 on the images including only DG regions. The average maximal value is 0.885 for *σ* = 5, and the *F*-score for every image including DG is superior to 0.850, which denotes a good quality of counting process. Values of *σ* between 4 and 6 provided high *F*-scores as well, which demonstrate the robustness of our choice.

The need to specifically process the DG is justified by the fact that it represents a particular case with a massive aggregation of small neurons. The size of the left DG on section No. 91 corresponds to 10^6^ pixels. In practice, the identification of the related connected components of DG is simple due to its size compared to other connected components that are significantly smaller.

### Comparison of Automated and Manual Neuron Counting

In terms of performance of the individualization of neurons, iCut, and Watershed were compared with the proposed method (for size-fixed neurons) in You et al., 2016. The results showed that our method gave the best individualization performance. However, due to the algorithmic complexity of iCut [*O(n^3^)*, where *n* is the number of foreground pixels in the image], we could not compare it with other methods on the individualization dataset. In addition, for the individualization of size-varying neurons, iCut is no longer adapted because a unique parameter for object size is fixed *a priori*. Therefore, we compared the proposed method with Watershed on the individualization dataset.

**FIGURE 8:**
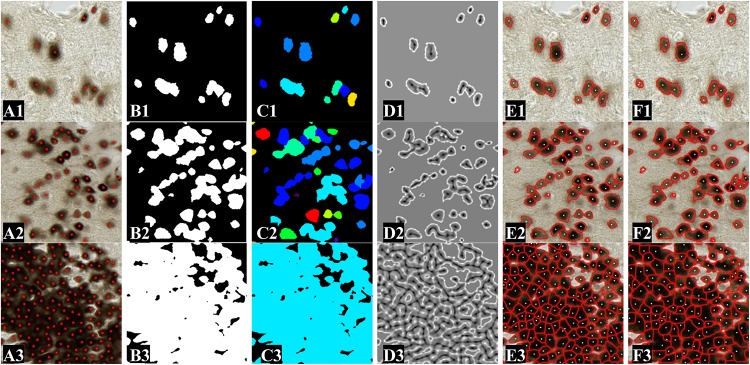
Neuron individualization results shown in images of 512 pixels × 512 pixels extracted from those obtained on the individualization dataset. Images 1–3 represent the anatomical region of thalamus, cortex, and DG, presenting different neuron densities. **(A1–A3)** Original images. Red points represent neuron centroids marked by the expert. **(B1–B3)** Binary images of segmented neuron class. **(C1–C3)** Images of optimal *σ*. **(D1–D3)** Min-max maps obtained after the two-step process of adaptive Gaussian filtering and min-max filtering. **(E1–E3)** Individualization results obtained using the proposed method. **(F1–F3)** Individualization results obtained using Watershed algorithm.

Figure 8 presents typical results on three different images presenting different neuron densities. Image 1 illustrates image of thalamus region with a few individual neurons, image 2 represents moderately dense image of cortex region in which several neurons touch each other, and image 3 represents extremely dense image of DG region in which many neurons are aggregated. Figures 8A1–A3 are the ground truth (red dots centroids manually annotated). Figures 8B1–B3 represent neuron classification results that are insufficient to segment individual neurons when aggregated neurons are present. Figures 8C1–C3 present the images of the optimal *σ* evaluated by the proposed method (local values estimated at neuron level). Figures 8D1–D3 present two-iteration min-max maps in which neuron centers appear in dark and neuron contours appear in bright-intensity values. Figures 8E1–E3 show the final individualization results obtained by the proposed method. Figures 8F1–F3 present the individualization results obtained by Watershed algorithm using our optimal *σ* map. Both methods perform well in simple cases. However, in more complex situations, Watershed results present overindividualization and under individualization, and making the segmented contours unnatural (straight borders, large neurons detected). Visually, the proposed method provides better neuron individualization results.

**FIGURE 9:**
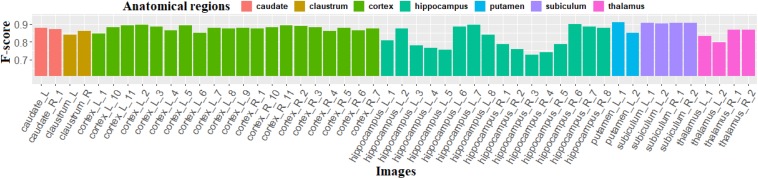
*F*-score calculated using our method on 50 images of the individualization dataset. L (respectively, R) means that this image is extracted from the left (respectively, right) side of the section.

Figure 9 shows the *F*-scores obtained with the proposed method for the 50 images of the individualization dataset demonstrating its ability to individualize the neurons with high *F*-scores, especially in the caudate, cortex, and subiculum, where average *F*-scores are, respectively, 0.874, 0.877, and 0.905. The *F*-scores computed in the hippocampus vary from 0.726 to 0.898 due to the heterogeneity of this region, which contains different kinds and distributions of neurons. Particularly, in the subregions CA2 and CA3, the intensity of certain neuron centers is lighter. These staining changes may be due to unsuitable staining marker (Mullen et al., 1992) corresponding to different levels of expression of the antigen. As the staining for this kind of neuron does not respect our hypothesis, the proposed method is not suitable in this case. All the same, the proposed method provides a good performance (*F*-score > 0.726).

The same evaluation was realized on the results produced using the Watershed algorithm. The mean *F*-score and standard deviation for seven anatomical regions are displayed in Table 3, showing that the proposed method systematically provides higher *F*-score values for every anatomical region. Moreover, the standard deviation of the proposed method is smaller, which demonstrates a good robustness for both simple and complex cases.

**TABLE 3:** *F*-scores computed using two neuron individualization methods, the proposed method and a Watershed algorithm.

**Anatomical regions**	**Proposed method**	**Watershed (optimal *σ*)**
Caudate	0.874 ± 0.003	0.791 ± 0.004
Claustrum	0.851 ± 0.015	0.762 ± 0.028
Cortex	0.877 ± 0.014	0.798 ± 0.020
Hippocampus	0.816 ± 0.062	0.687 ± 0.080
Putamen	0.881 ± 0.042	0.813 ± 0.039
Subiculum	0.905 ± 0.001	0.831 ± 0.006
Thalamus	0.841 ± 0.034	0.736 ± 0.039

### Study of the Location of the Centroids and the Contours of the Neurons

The quality of the individualization process between the proposed method and the Watershed algorithm using the optimal *σ* map was compared.

**FIGURE 10:**
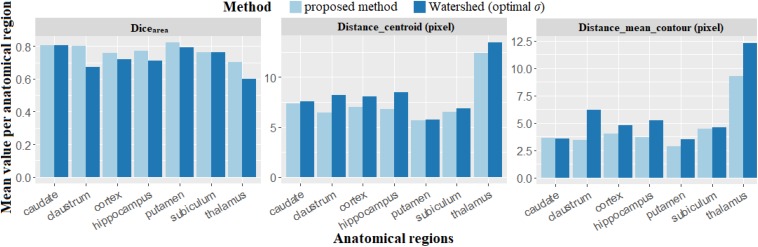
Results obtained to compare the different individualization methods, the proposed method and Watershed using new criteria (*Dice*_*area*_, *Distance_centroid* and *Distance_mean_contour*). *Dice*_*area*_ represents the overlapping fraction between neurons automatically and manually individualized, *Distance_mean_contour* and *Distance_centroid* represent, respectively, the distances of the contours and the centroids between automated and manual individualization results.

Figure 10 presents a comparison synthesis of the results for the supplementary parameters investigated. It shows that neurons segmented by the proposed method superimpose better with the neurons segmented manually than those segmented with Watershed. The average value of *Dice*_*area*_ is 0.77 for the proposed method and 0.72 for Watershed. The detected centroids and segmented contours, compared to Watershed, are closer to those segmented manually by the expert. The average value of the distance between centroids (*Distance_centroid*) and of the distance between contours (*Distance_mean_contour*) are, respectively, 1.31 and 0.98 μm for the proposed method, and 1.45 and 1.26 μm for Watershed. Again, we observe that the quality of individualization of neurons using our method is superior compared to the one obtained with Watershed. These quantitative indexes are of major interest because they resume and confirm the qualitative evaluation of the individualization previously mentioned in Figure 9. They provide additional quantitative local and spatial information concerning neuron individualization quality.

### Comparison of Automated Neuron Counting Versus Stereology

**FIGURE 11:**
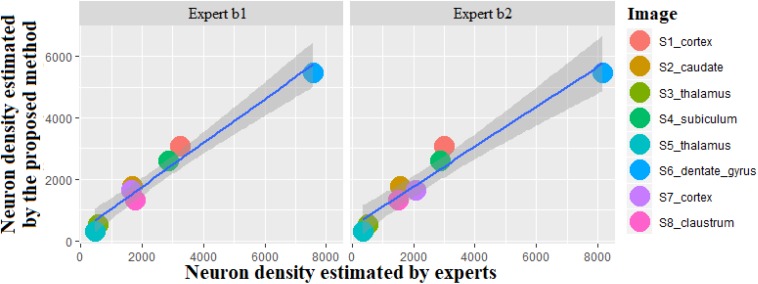
Scatterplot of neuron density calculated by the proposed method compared with stereological estimations produced by the two experts. The unit is the number of neurons per square millimeter.

We validated the proposed method by comparing our results with an adapted stereology technique using the method of the optical dissectors, which is the gold standard method in biology to count objects. No dead zones were considered for stereology technique to enable comparison in the same conditions (no depth information available in image processing approach). This technique is operator-dependent, two experts (b1 and b2) performed the counting twice by modifying the sampling fraction to obtain a stable estimation of the number of neurons and ensured the high counting accuracy. In this study, the sampling fraction varied from 1/100 to 1/4 on eight large images. The results obtained with stereology using the second sampling were selected (Supplementary Table 4). As the surface of eight images are very different, neuron density instead of neuron number was considered to normalize the results obtained using the proposed method and the adapted stereology technique. The analysis was performed with linear regression shown in Figure 11. The dark gray color represents the confidence interval around the solid line in dark blue color. The correlation coefficient between neuron densities was computed with the proposed method and the adapted stereology technique performed by two experts (b1 and b2). The high correlation values (0.983 for b1 and 0.975 for b2) and the low p-value (1.20e-05 for b1 and 3.76e-05 for b2) state that the proposed method is very promising to estimate the neuron density and the number of neurons in different anatomical regions.

### Average Neuron Radius in Different Anatomical Regions

**FIGURE 12:**
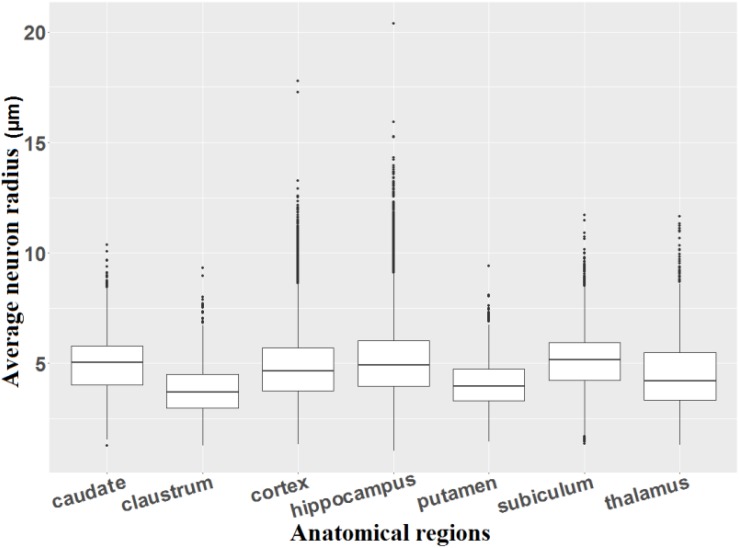
Average radius of individualized neurons.

Figure 12 shows the average radius of individualized neurons in different anatomical regions of the macaque brain. Six summary statistics (minimum, first quartile, average, median, third quartile, and maximum) are listed in Supplementary Table 5.

We observe that most of the neurons, which remain in the range of the first and third quartiles, in the claustrum (average radius ranging from 2.99 to 4.52 μm) and the putamen (average radius varying from 3.31 to 4.73 μm) are smaller than the neurons of the other regions (average radius varying from 3.34 to 6.02 μm). The average radius of individualized neurons in our study varies from 1 to 14.3 μm (corresponding from 2 to 28.6 μm in diameter, five outliers for neurons were excluded), which corresponds quite well to the study by Andersen et al. (2016) who estimated that the size of neurons varies between 5 and 30 μm in diameter. The detection of the very small neurons may be due to the production of partial neurons during the cutting process or to a problem during the digitization (focal plane of scanning not centered on the neuron, position of the neurons in the depth of the cut, damaged cells during staining, etc.).

### Execution Time

The proposed method was implemented in C++ on a 64-bit computer workstation under Linux (CPU: Intel Xeon E5-2630 v3 at 2.4 GHz, RAM: 128 GB). Twenty-three cores of the computer were used for the parallel calculation of 58 images of the individualization dataset [BrainVISA Soma-Workflow module (Laguitton et al., 2011), CPU parallelization]. The average execution time for the final individualization result was 15.6 h, that is, 16.1 min per image of 5,000 pixels × 5,000 pixels. This execution time is very reasonable compared to the necessary time to do stereology, and it is perfectly adapted to deal with large images especially with parallelization.

## Discussion

We proposed a novel method for the individualization of size-varying and large number of touching neurons in microscopic macaque brain image. We first applied an RF classifier, a reliable segmentation approach, based on four features (L^∗^, M, V, and LBP40) to segment stained and non-stained tissue. This combination of features presented a limited number of color and texture features, which give satisfactory segmentation results. Alternative methods can also be considered and plugged into our protocol. In particular, convolutional neural networks (CNN)-based methods are promising approaches. As a preliminary test, we applied U-net (Ronneberger et al., 2015) on the segmentation dataset, and we obtained comparable segmentation results. To individualize the touching size-varying neurons, we model each neuron individually (centroid and contour) to deal with different sizes and densities. The proposed method investigates all possible individualization results parameterized by *σ*, which defines the size of the Gaussian kernel filter, and then determines the optimal *σ* for each NOI based on the Dice score. Using this adaptive Gaussian filter and an original min-max filter, the key information such as location (centroid) and boundary of each expected NOI is enhanced in a map of extrema. The multiple studies performed in the present work confirmed the genericity and interest to use the min-max filter. Finally, a discrete contour-based model is applied to achieve neuron individualization. This model is a robust region-based segmentation method. Instead of examining all neighboring pixels of initial seeds used in traditional region growing methods, this model examines several pixels of discrete contour. Taking advantage of information about pixel intensity in the extrema map and contour curvature at the pixel level, discrete contours expand through a competition approach. Besides, the use of discrete contour alleviates the noise on the neuron contour finally segmented and saves execution time. Considering the general satisfactory results obtained by U-net on other biological data, a future work will be initiated soon to test the ability of U-net to individualize touching cell as it was recently proposed (Falk et al., 2019).

The proposed method provides good performances both qualitatively and quantitatively. It is worth mentioning that our method is able to handle the most difficult cases involving massive touching neurons like in the DG, which is part of the hippocampus. This region of the hippocampus is extremely complex to analyze. An interesting biological result is that the size of the neurons in DG regions is stable based on the single optimal value of *σ* estimated (Figure 7). The hippocampus contains a wide range of neurons with different sizes and different kinds of neurons in several subregions (CA2 and CA3), which can lead to different staining and non-optimal individualization results. The *F*-scores in the CA2 and CA3 vary between 0.726 and 0.788. Nevertheless, the mean *F*-score still reaches 0.816 in the hippocampus, denoting that several subregions (CA1, CA4, and DG) of the hippocampus can be successfully treated by the proposed method. Most of the biological studies aiming to count neurons avoid to target this region due to its complexity and favor optic density for quantitative measurements (Brureau et al., 2017).

The proposed method was compared with Watershed, a reference individualization method in the literature. In addition to the *F*-score described above, three supplementary criteria were proposed in this article: mean distance between the centroids, mean distance between the contours, and Dice superimpositions. According to these criteria, the neurons individualized by the proposed method are better superimposed with manual segmentation results than Watershed (0.77 for the proposed method vs. 0.72 for Watershed). Moreover, the centroids and contours segmented by the proposed method are closer to those segmented by the experts (respectively, 9.98 and 21.89% closer) than Watershed. Therefore, the proposed method is proven to be an efficient method to properly identify centroids and accurately estimate the location of neuron contours. All datasets produced in this study will be made available to enable fair benchmarking.

To further validate the proposed method, we also applied stereology, the gold standard in the domain, onto a dedicated dataset with the participation of the two experts. They were asked to estimate the number of neurons in various anatomical ROIs, which were selected in accordance with their interest in biology. Therefore, we obtained a wide range of neuron densities present in the brain (ranging from 343 to 8,167 neurons per mm^2^). The neuron density calculated by the proposed method is highly correlated with those estimated by the stereology technique (0.983 and 0.975 respectively, for b1 and b2 experts). In this work, the dead zones classically used in stereology that exclude cut cells were not considered image processing approaches and cannot deal with this phenomenon (Z-stack can be envisioned but will dramatically increase the amount of data to deal with). In this situation, the results obtained by the experts and by our method were consistent. Nevertheless, it will be necessary to precisely estimate the bias introduced at this occasion to evaluate, based on a tolerance level to be defined, if our method could be or not an alternative to stereology in biological studies. This point is the main limitation of the proposed method compared to stereology. On the other hand, our method makes it possible to rapidly analyze large amounts of data, which can provide a first screening at brain-section level to identify and investigate regions of interest. Such a strategy is hardly feasible with stereology. This work is a preliminarily study in major anatomical ROIs on a single histological section and is certainly worth being extended to study entire brains (series of histological sections) and multiple animals (Amunts et al., 2013). Future work will also aim to evaluate the effects of staining variations as well as intersubject effects in our protocol. These preliminary results demonstrate the potential of the proposed method to individualize and count the neurons with high accuracy. To our knowledge, this is the first study in this field to use stereology to evaluate an automated analysis method.

The validation work performed in this study constitutes an original contribution and produced original processed data. In total, five experts participated in this time-consuming and tedious work. Four reference databases annotated by experts were derived from this study and can be used for future research and development: (1) manual segmentation of neuronal staining in 100 images (512 pixels × 512 pixels) in the segmentation dataset, (2) 111,971 neuron centroids manually marked in the individualization dataset concerning 50 images (5,000 pixels × 5,000 pixels), (3) 900 neuron centroids and contours drawn by experts in the individualization dataset concerning nine images, and (4) 37,000 neurons counted by experts using stereology technique in the stereology dataset.

With the ability to count neurons of the proposed method, it is possible to extend neuron counting to series of sections or to an entire brain. For instance, the section 91 consists of about 230,000 pixels × 188,000 pixels. We can divide this section into about 1,730 images of 5,000 pixels × 5,000 pixels. These images can be processed in parallel by the proposed method. The number of neurons in one section can then be calculated and mesoscopic quantitative heat maps of neuron density derived (Vandenberghe et al., 2016). The use of a supercomputer can dramatically decrease computation time, making it possible to significantly extend this study to produce original cartographies of neuron distribution through the brain.

Preliminary results of parallelization of segmentation codes have demonstrated a high scalability of our method. This work is carried out on a CPU-based architecture but may be extended to GPU architectures in the future, particularly with regard to the implementation of deep learning methods.

A first application aiming to compute neuron size was performed on the individualization dataset. Although neurons have various shapes and may be cut according to their location in the histological section, we can still estimate the average neuron radius with statistical tools, thanks to the large number of neurons processed. This information can be used to roughly estimate neuron size or to perform comparative studies considering that similar biases are introduced in various measurements. The statistical result shows that neuron size calculated by the proposed method (2–28.6 μm in diameter) fits well with the size that was read in the literature (5–30 μm in diameter). It is important to keep in mind that morphological measurements made in 2-D remain an estimate and that it is necessary to acquire volumes to obtain more precise measurements. In this context, the first strength of our approach is the new extracted information to enrich current knowledge of the available data. For instance, the proposed individualization method provides interesting descriptive information (location, size, intensity, shape, and network organization) on the population of neurons for each region. In anatomical regions, this information can be used to better describe brain development, aging, disease, or even evaluate a therapy by producing analyses at the cellular level. The second strength of our approach is the scalability of these technologies (to whole section, brain, and groups) as all processings are performed at the connected component level, which makes parallelization schemes extremely efficient. It will support interesting perspectives for biological studies targeting cytoarchitecture analysis.

Nowadays, neuron individualization is still an important and challenging subject of research in the field of neuroscience. In 2014, Schmitz et al. (2014) compared available methods for cell segmentation, showing that the individualization of cells is a complex problem and stressing the need to develop new approaches in this area. Although several works have been done over the past years, most of the proposed methods are based on mathematical morphology, detection of concavities, the Watershed algorithm, and graph-cut algorithms. Deep learning is a promising approach in the future but needs to be carefully investigated to evaluate its ability to robustly solve this question. Most of these methods have been focused on relatively simple or very specific cases. When we deal with complex cases, such as the DG, where thousands of neurons are aggregated, these methods cannot properly individualize neurons. In fact, the reason why the existing image processing methods do not perform well is that they are performed on 2-D images, with a single scanning focus plane, which increases the difficulty of individualizing touching neurons. It might be possible to acquire several planes associated to the depth in the sections when scanning using Z-stack technique so as to reconstruct a pseudo-3-D volume and apply an improved version of the proposed method. However, new problems would inevitably arise, in particular the increase of the volumetry of the data to deal with.

Nevertheless, this work is promising. The proposed tool can help address major biological challenges, such as improving our understanding of brain development or aging, deciphering pathology mechanisms, or evaluating novel therapies in neurodegenerative diseases.

## Conclusion

This paper presents a robust and efficient method to individualize size-varying and touching neurons in microscopic images of macaque brain sections. The results obtained are promising. The accuracy of the proposed method is close to 0.816 in the hippocampus, which is a very complex anatomical region and superior to 0.841 in the other regions studied (caudate, claustrum, cortex, putamen, subiculum, and thalamus). Moreover, the accuracy and performance of the proposed method are significantly higher qualitatively and quantitatively compared to Watershed algorithm in the anatomical regions tested. Compared with an adapted stereology technique, we found that the counting results obtained with our method are highly correlated (0.983 for b1 and 0.975 for b2) and could be considered as a reliable alternative. Supplementary researches based on extended datasets (sections and animals) should be envisioned to confirm this point. The average neuron radius estimated by the proposed method is coherent compared to the acknowledged findings of literature. The automated detection of millions of neurons in the whole brain is still challenging. Perspectives of this work will be to extend the application of the proposed method to entire brains.

## Data Availability Statement

The datasets generated for this study will not be made publicly available. The data will be available later in the form of an article and an access because it corresponds to a large amount of information.

## Ethics Statement

The animal study was reviewed and approved by the Comité d’éthique agréé par le MESR dont relève l’EU: CETEA DSV – Comité n°44.

## Author Contributions

ZY, CJ, A-SH, and TD created the datasets. ZY, YB, CB, NS, and TD developed the software and performed the image processing. A-SH and TD performed the tissue classification. ZY and TD located the neuron centroids and drew the neuron contours. PG and CJ estimated the neuron number with stereological techniques. ZY, A-SH, and TD performed the statistical analysis. A-SH, PG, CJ, PH, and TD planned the histology and imaging experiments. ZY and TD wrote the manuscript.

## Conflict of Interest

The authors declare that the research was conducted in the absence of any commercial or financial relationships that could be construed as a potential conflict of interest.
