# Supplementary material for: Automated Individualization of Size-Varying and Touching Neurons in Macaque Cerebral Microscopic Images
Source: Front Neuroanat. 2019 Dec 17;13:98. doi: 10.3389/fnana.2019.00098 (PMC6929681; doi:10.3389/fnana.2019.00098)
Supplement: Supplementary file 1 [file Data_Sheet_1.PDF]

frontiers

Supplementary Material

1 Major anatomical regions studied in this work

Supplementary Table 1. List of the major anatomical regions used in three different datasets. Se: *segmentation dataset*, I: *individualization dataset*, St: *stereology dataset*.

N°	Anatomical regions		Dataset
	Major regions	Sub-regions	
1	Cortex	Cortex layer 1-6	Se / I / St
2	Caudate	/	Se / I / St
3	Thalamus	/	Se / I / St
4	Claustrum	/	Se / I / St
5	Putamen	/	Se / I
6	Hippocampus	CA1-4 (Cornu Ammonis 1-4)	Se / I
		Dentate gyrus	Se / I / St
7	Subiculum	Subiculum	Se / I / St
		Para-subiculum	
8	Globus pallidus	Globus pallidus internal	Se
		Globus pallidus external	
9	Hypothal (Hypothalamus)	/	Se
10	Habenula	/	Se
11	Mammillary body	/	Se
12	LG (lateral geniculate)	/	Se
13	PAG (periaqueductal gray)	/	Se
14	Pons	/	Se
15	White matter	/	Se / I
16	Ventricle	/	Se / I
17	Background	/	Se

2 Dataset for the segmentation of neuronal staining – *segmentation dataset*

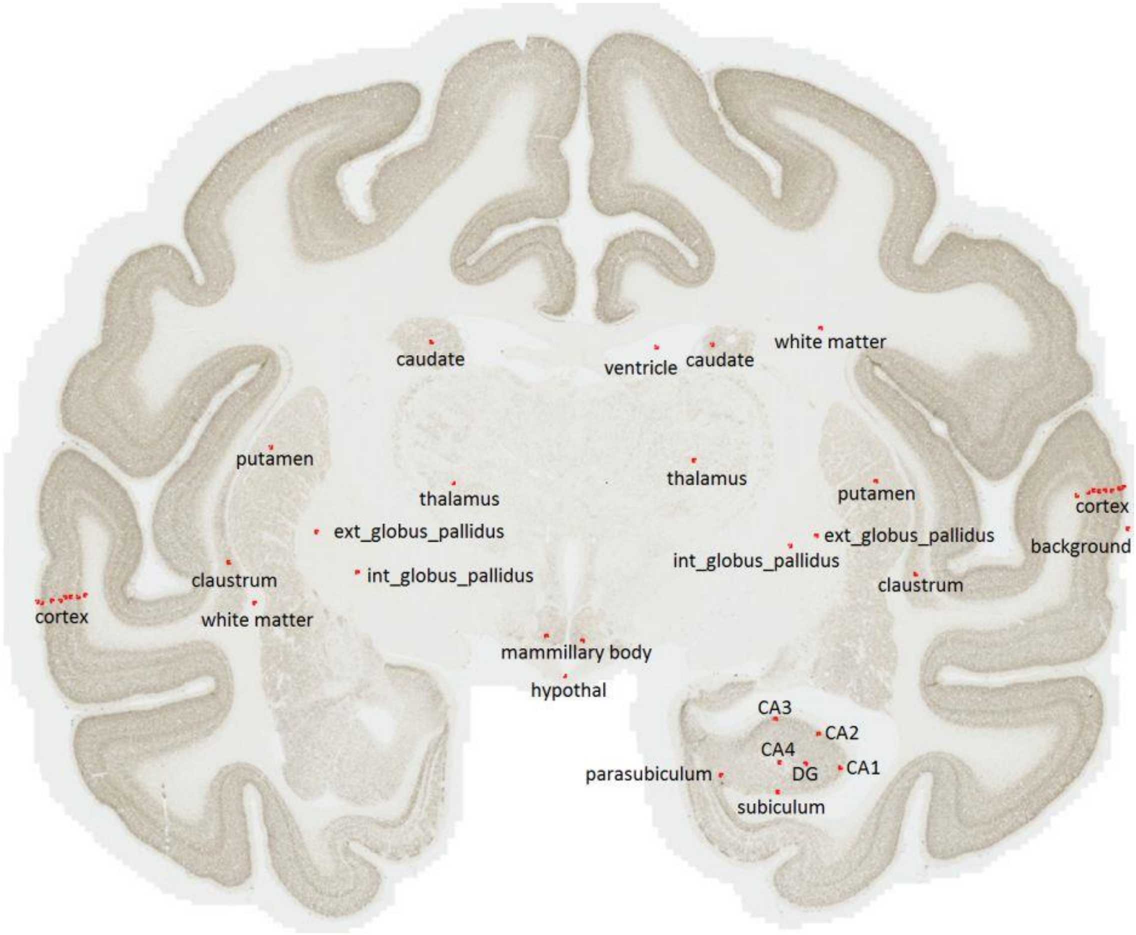

Supplementary Figure 1. Image of brain section N° 81 of 216,000x178,000 pixels (47.57 mm x 39.16 mm), ~ 130 GB. The red squares represent the spatial location of images of 512x512 pixels in the *segmentation dataset*. The legends correspond to anatomical structures to which they belong.

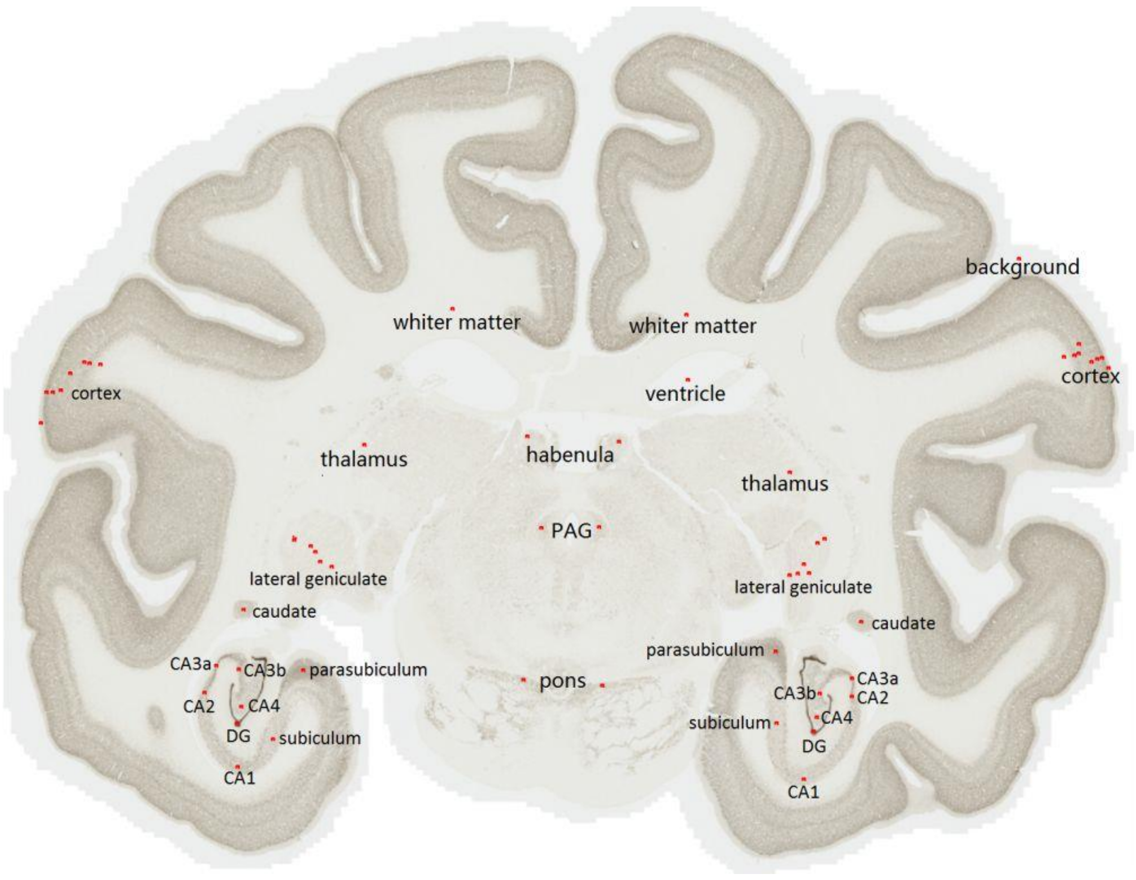

Supplementary Figure 2. Image of brain section N° 101 of 236,000x181,000 pixels (52 mm x 39.9 mm), ~ 148 GB. The red squares represent the spatial location of images of 512x512 pixels in the *segmentation dataset*. The legends correspond to anatomical structures to which they belong.

Supplementary Table 2. List of *segmentation dataset* images for the segmentation of neuronal staining. Side: L – Left, R – Right, C – central. L/V: Learning set / Validation set. The size of images is 512x512 pixels.

N°	Image name	Side	Region	Position X	Position Y	L/V
1	CJ1301_slide81unsharp_BG_R	R	background	215024	100457	L
2	CJ1301_slide81unsharp_CA1_R	R	CA1	160066	146153	L
3	CJ1301_slide81unsharp_CA2_R	R	CA2	155891	139728	L
4	CJ1301_slide81unsharp_CA3_R	R	CA3	147641	136853	L
5	CJ1301_slide81unsharp_CA4_R	R	CA4	148456	145163	L
6	CJ1301_slide81unsharp_caudate_L	L	caudate	81930	64713	V
7	CJ1301_slide81unsharp_caudate_R	R	caudate	135517	65280	L
8	CJ1301_slide81unsharp_claustrum_L	L	claustrum	43161	106864	V

9	CJ1301_slide81unsharp_claustrum_R	R	claustrum	174451	109137	L
10	CJ1301_slide81unsharp_ctx_L_layer1	L	cortex layer1	6538	114411	V
11	CJ1301_slide81unsharp_ctx_L_layer2	L	cortex layer2	7375	114628	L
12	CJ1301_slide81unsharp_ctx_L_layer3	L	cortex layer3	9386	114274	V
13	CJ1301_slide81unsharp_ctx_L_layer4	L	cortex layer4	10962	114238	V
14	CJ1301_slide81unsharp_ctx_L_layer5a	L	cortex layer5a	11864	113558	L
15	CJ1301_slide81unsharp_ctx_L_layer5b	L	cortex layer5b	12980	113494	L
16	CJ1301_slide81unsharp_ctx_L_layer6a	L	cortex layer6a	14313	113494	L
17	CJ1301_slide81unsharp_ctx_L_layer6b	L	cortex layer6b	15736	113172	V
18	CJ1301_slide81unsharp_ctx_R_layer1	R	cortex layer1	214234	92503	L
19	CJ1301_slide81unsharp_ctx_R_layer2	R	cortex layer2	213394	92727	V
20	CJ1301_slide81unsharp_ctx_R_layer3	R	cortex layer3	211980	93207	L
21	CJ1301_slide81unsharp_ctx_R_layer4	R	cortex layer4	210620	93159	L
22	CJ1301_slide81unsharp_ctx_R_layer5a	R	cortex layer5a	209532	93303	V
23	CJ1301_slide81unsharp_ctx_R_layer5b	R	cortex layer5b	208508	93127	V
24	CJ1301_slide81unsharp_ctx_R_layer6a	R	cortex layer6a	207388	93463	V
25	CJ1301_slide81unsharp_ctx_R_layer6b	R	cortex layer6b	205276	94103	L
26	CJ1301_slide81unsharp_DG_R	R	dentate gyrus	153366	145403	L
27	CJ1301_slide81unsharp_globus_pallidus_ext_L	L	globus pallidus	59954	101070	V
28	CJ1301_slide81unsharp_globus_pallidus_ext_R	R	globus pallidus	155441	101799	L
29	CJ1301_slide81unsharp_globus_pallidus_int_L	L	globus pallidus	67746	108801	L
30	CJ1301_slide81unsharp_globus_pallidus_int_R	R	globus pallidus	150434	103746	V
31	CJ1301_slide81unsharp_hypothal	C	hypothal	107307	128570	L
32	CJ1301_slide81unsharp_mam_body_L	L	mammillary_body	103854	120974	L
33	CJ1301_slide81unsharp_mam_body_R	R	mammillary_body	110739	121899	V
34	CJ1301_slide81unsharp_putamen_L	L	putamen	51147	84883	L
35	CJ1301_slide81unsharp_putamen_R	R	putamen	166812	91399	V
36	CJ1301_slide81unsharp_subiculum_para_R	R	para-subiculum	137257	147603	L
37	CJ1301_slide81unsharp_subiculum_R	R	subiculum	148216	150803	L
38	CJ1301_slide81unsharp_thalamus_L	L	thalamus	86018	91673	L
39	CJ1301_slide81unsharp_thalamus_R	R	thalamus	132095	87392	V
40	CJ1301_slide81unsharp_ventricle_R	R	ventricle	124922	65719	V

41	CJ1301_slide81unsharp_WM_L	L	white matter	48104	114674	V
42	CJ1301_slide81unsharp_WM_R	R	white matter	156246	62111	L
43	CJ1301_slide101unsharp_BG_R	R	background	212245	52644	V
44	CJ1301_slide101unsharp_CA1_L	L	CA1	49025	158817	L
45	CJ1301_slide101unsharp_CA1_R	R	CA1	167285	161394	V
46	CJ1301_slide101unsharp_CA2_L	L	CA2	42084	143295	L
47	CJ1301_slide101unsharp_CA2_R	R	CA2	177325	144103	V
48	CJ1301_slide101unsharp_CA3a_L	L	CA3a	44549	137657	L
49	CJ1301_slide101unsharp_CA3a_R	R	CA3a	177356	140325	V
50	CJ1301_slide101unsharp_CA3b_L	L	CA3b	49255	138429	V
51	CJ1301_slide101unsharp_CA3b_R	R	CA3b	170600	143485	L
52	CJ1301_slide101unsharp_CA4_L	L	CA4	49763	146237	L
53	CJ1301_slide101unsharp_CA4_R	R	CA4	169976	148447	V
54	CJ1301_slide101unsharp_caudate_L	L	caudate	50258	125958	L
55	CJ1301_slide101unsharp_caudate_R	R	caudate	179257	128488	V
56	CJ1301_slide101unsharp_ctx_L_layer1	L	cortex layer1	7920	86958	L
57	CJ1301_slide101unsharp_ctx_L_layer2	L	cortex layer2	9075	80531	V
58	CJ1301_slide101unsharp_ctx_L_layer3	L	cortex layer3	10405	80533	L
59	CJ1301_slide101unsharp_ctx_L_layer4	L	cortex layer4	12079	80133	L
60	CJ1301_slide101unsharp_ctx_L_layer5a	L	cortex layer5a	14044	76592	V
61	CJ1301_slide101unsharp_ctx_L_layer5b	L	cortex layer5b	16993	74252	V
62	CJ1301_slide101unsharp_ctx_L_layer6a	L	cortex layer6a	18092	74491	V
63	CJ1301_slide101unsharp_ctx_L_layer6b	L	cortex layer6b	20348	74728	L
64	CJ1301_slide101unsharp_ctx_R_layer1	R	cortex layer1	230933	75485	V
65	CJ1301_slide101unsharp_ctx_R_layer2	R	cortex layer2	229580	73350	L
66	CJ1301_slide101unsharp_ctx_R_layer3	R	cortex layer3	228587	73598	V
67	CJ1301_slide101unsharp_ctx_R_layer4	R	cortex layer4	227383	74206	V
68	CJ1301_slide101unsharp_ctx_R_layer5a	R	cortex layer5a	224744	70457	L
69	CJ1301_slide101unsharp_ctx_R_layer5b	R	cortex layer5b	224692	72436	L
70	CJ1301_slide101unsharp_ctx_R_layer6a	R	cortex layer6a	223797	72825	L
71	CJ1301_slide101unsharp_ctx_R_layer6b	R	cortex layer6b	221626	73142	V
72	CJ1301_slide101unsharp_DG_L	L	dentate gyrus	48819	149736	L

73	CJ1301_slide101unsharp_DG_R	R	dentate gyrus	169343	151390	V
74	CJ1301_slide101unsharp_habenula_L	L	habenula	109521	89758	V
75	CJ1301_slide101unsharp_habenula_R	R	habenula	128654	90860	L
76	CJ1301_slide101unsharp_LG_L_1	L	LG	68660	116983	L
77	CJ1301_slide101unsharp_LG_L_2	L	LG	66256	115925	V
78	CJ1301_slide101unsharp_LG_L_3	L	LG	65241	113883	L
79	CJ1301_slide101unsharp_LG_L_4	L	LG	64272	112680	V
80	CJ1301_slide101unsharp_LG_L_5	L	LG	60837	111409	L
81	CJ1301_slide101unsharp_LG_L_6	L	LG	60914	110889	V
82	CJ1301_slide101unsharp_LG_R_1	R	LG	164301	118741	V
83	CJ1301_slide101unsharp_LG_R_2	R	LG	166026	118416	L
84	CJ1301_slide101unsharp_LG_R_3	R	LG	167298	116469	V
85	CJ1301_slide101unsharp_LG_R_4	R	LG	168376	118300	L
86	CJ1301_slide101unsharp_LG_R_5	R	LG	170150	112041	L
87	CJ1301_slide101unsharp_LG_R_6	R	LG	171612	111158	V
88	CJ1301_slide101unsharp_PAG_L	L	PAG	112445	108877	V
89	CJ1301_slide101unsharp_PAG_R	R	PAG	124524	108709	L
90	CJ1301_slide101unsharp_pons_L	L	pons	108715	140626	L
91	CJ1301_slide101unsharp_pons_R	R	pons	125184	141824	V
92	CJ1301_slide101unsharp_subiculum_para_L	L	para-subiculum	62704	138606	L
93	CJ1301_slide101unsharp_subiculum_para_R	R	para-subiculum	161407	134685	V
94	CJ1301_slide101unsharp_subiculum_L	L	subiculum	56393	153016	L
95	CJ1301_slide101unsharp_subiculum_R	R	subiculum	161588	149664	V
96	CJ1301_slide101unsharp_thalamus_L	L	thalamus	75488	91524	V
97	CJ1301_slide101unsharp_thalamus_R	R	thalamus	164357	97270	L
98	CJ1301_slide101unsharp_ventricle_R	R	ventricle	143068	77944	L
99	CJ1301_slide101unsharp_WM_L	L	white matter	93897	63118	L
100	CJ1301_slide101unsharp_WM_R	R	white matter	142766	64349	V

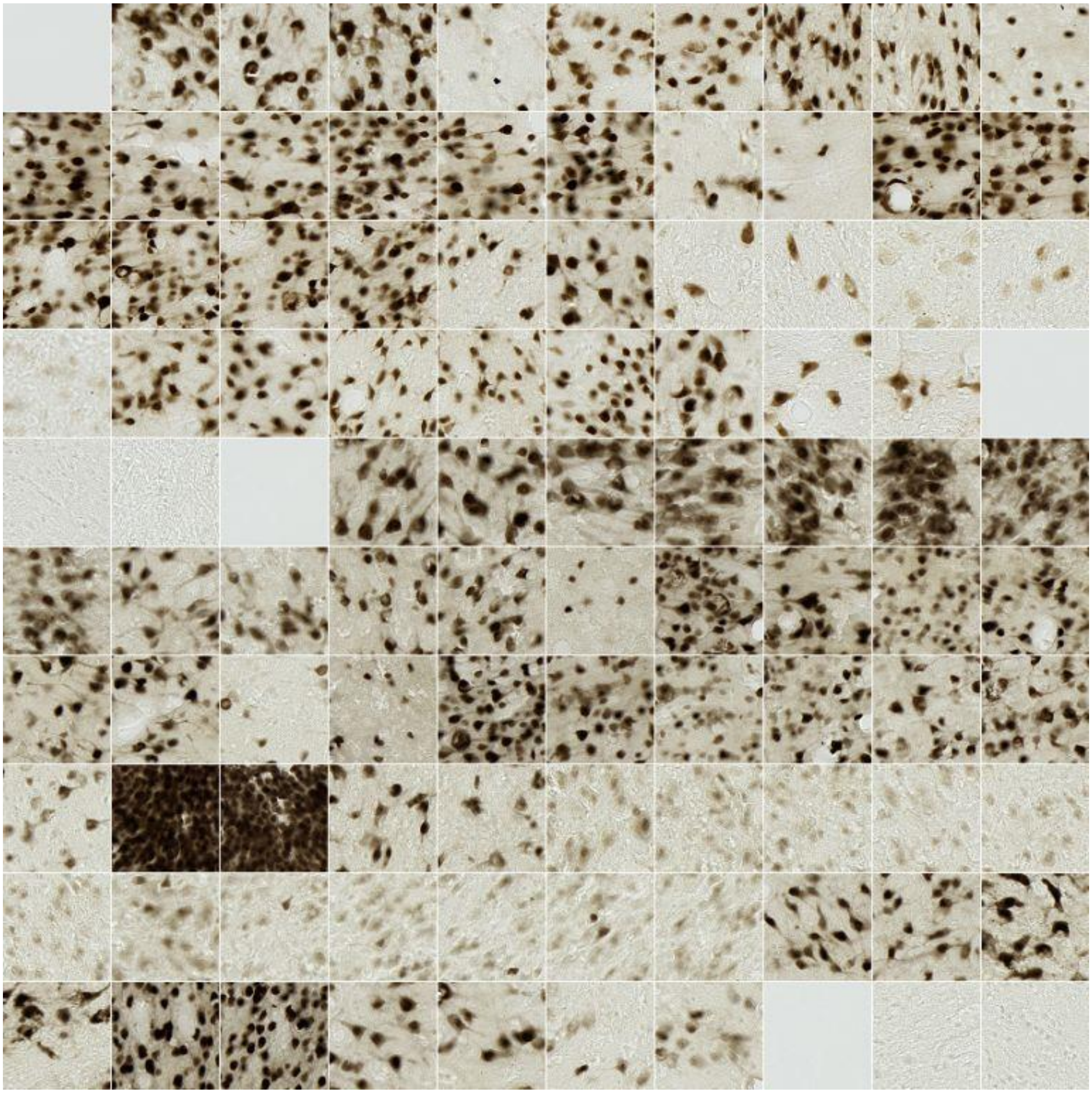

Supplementary Figure 3. *Segmentation dataset* for the segmentation of neuronal staining. From left to right, from top to bottom: image number is from N° 1 to N° 100.

3 Dataset for the individualization of neurons – *individualization dataset*

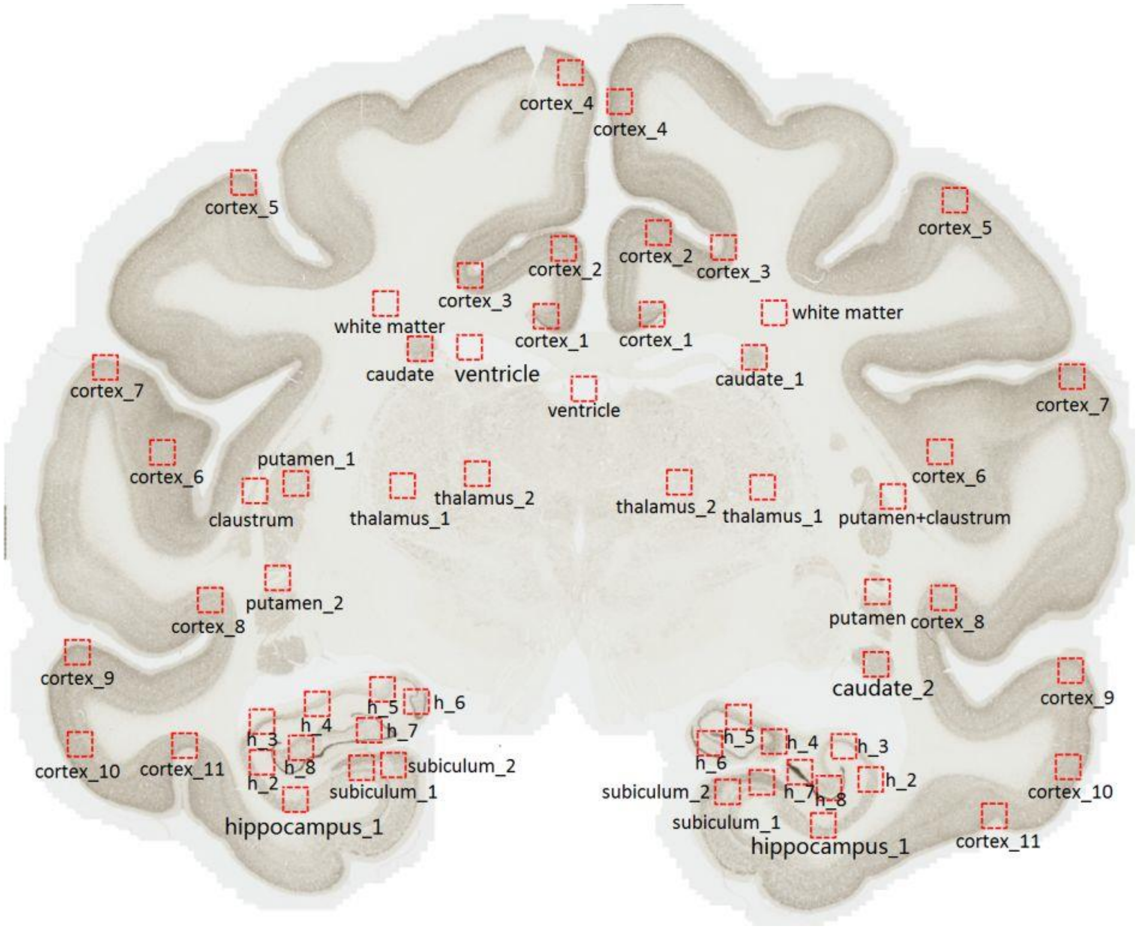

Supplementary Figure 4. Image of brain section N° 91 of 230,000x188,000 pixels (50.74 mm x 41.33 mm), ~ 145 GB. The red squares represent the spatial location of images of 5000x5000 pixels in the *individualization dataset*. "h" is an abbreviation for the name of the region of the hippocampus. The numbers at the end of each region corresponds to the number of the images in these regions.

Supplementary Table 3. List of *individualization dataset* images for the individualization of neurons. Side: L – Left, R – Right, C – Central. The size of images is 5000x5000 pixels. Thereinto, the images of N° 3, 24, 25 and 46 are so fuzzy that expert cannot distinguish neurons.

N°	Image name	Side	Region	Position X	Position Y
1	CJ1301_slide91unsharp_caudate_L	L	caudate	82522	67838
2	CJ1301_slide91unsharp_caudate_R_1	R	caudate	150612	69601
3	CJ1301_slide91unsharp_caudate_R_2	R	caudate	175410	132107
4	CJ1301_slide91unsharp_claustrum_L	L	claustrum	48891	96789

5	CJ1301_slide91unsharp_claustrum_R	R	claustrum	178852	97930
6	CJ1301_slide91unsharp_ctx_L_1	L	cortex	108174	61247
7	CJ1301_slide91unsharp_ctx_L_2	L	cortex	111775	47451
8	CJ1301_slide91unsharp_ctx_L_3	L	cortex	92715	52930
9	CJ1301_slide91unsharp_ctx_L_4	L	cortex	113133	11625
10	CJ1301_slide91unsharp_ctx_L_5	L	cortex	46576	34018
11	CJ1301_slide91unsharp_ctx_L_6	L	cortex	30036	88960
12	CJ1301_slide91unsharp_ctx_L_7	L	cortex	18414	71644
13	CJ1301_slide91unsharp_ctx_L_8	L	cortex	39760	119015
14	CJ1301_slide91unsharp_ctx_L_9	L	cortex	12737	129645
15	CJ1301_slide91unsharp_ctx_L_10	L	cortex	13244	148385
16	CJ1301_slide91unsharp_ctx_L_11	L	cortex	34526	148654
17	CJ1301_slide91unsharp_ctx_R_1	R	cortex	129792	60821
18	CJ1301_slide91unsharp_ctx_R_2	R	cortex	131056	44222
19	CJ1301_slide91unsharp_ctx_R_3	R	cortex	144342	47234
20	CJ1301_slide91unsharp_ctx_R_4	R	cortex	123157	17547
21	CJ1301_slide91unsharp_ctx_R_5	R	cortex	191482	37605
22	CJ1301_slide91unsharp_ctx_R_6	R	cortex	188387	88838
23	CJ1301_slide91unsharp_ctx_R_7	R	cortex	215311	73538
24	CJ1301_slide91unsharp_ctx_R_8	R	cortex	189168	118593
25	CJ1301_slide91unsharp_ctx_R_9	R	cortex	215041	133395
26	CJ1301_slide91unsharp_ctx_R_10	R	cortex	214521	153002
27	CJ1301_slide91unsharp_ctx_R_11	R	cortex	199475	162973
28	CJ1301_slide91unsharp_hip_L_1	L	hippocampus	57130	159664
29	CJ1301_slide91unsharp_hip_L_2	L	hippocampus	50320	152057
30	CJ1301_slide91unsharp_hip_L_3	L	hippocampus	50324	143779
31	CJ1301_slide91unsharp_hip_L_4	L	hippocampus	61567	140170
32	CJ1301_slide91unsharp_hip_L_5	L	hippocampus	74853	137205
33	CJ1301_slide91unsharp_hip_L_6	L	hippocampus	81808	139695
34	CJ1301_slide91unsharp_hip_L_7	L	hippocampus	72156	145516
35	CJ1301_slide91unsharp_hip_L_8	L	hippocampus	58265	149115
36	CJ1301_slide91unsharp_hip_R_1	R	hippocampus	164647	164902

37	CJ1301_slide91unsharp_hip_R_2	R	hippocampus	174314	155545
38	CJ1301_slide91unsharp_hip_R_3	R	hippocampus	168837	148803
39	CJ1301_slide91unsharp_hip_R_4	R	hippocampus	154655	147824
40	CJ1301_slide91unsharp_hip_R_5	R	hippocampus	147370	142851
41	CJ1301_slide91unsharp_hip_R_6	R	hippocampus	141545	148185
42	CJ1301_slide91unsharp_hip_R_7	R	hippocampus	159898	154049
43	CJ1301_slide91unsharp_hip_R_8	R	hippocampus	165822	157309
44	CJ1301_slide91unsharp_putamen_L_1	L	putamen	57213	95283
45	CJ1301_slide91unsharp_putamen_L_2	L	putamen	53517	114512
46	CJ1301_slide91unsharp_putamen_R	R	putamen	175690	117341
47	CJ1301_slide91unsharp_subiculum_L_1	L	subiculum	70588	153176
48	CJ1301_slide91unsharp_subiculum_L_2	L	subiculum	77069	152620
49	CJ1301_slide91unsharp_subiculum_R_1	R	subiculum	152133	156150
50	CJ1301_slide91unsharp_subiculum_R_2	R	subiculum	145144	158084
51	CJ1301_slide91unsharp_thalamus_L_1	L	thalamus	78962	95737
52	CJ1301_slide91unsharp_thalamus_L_2	L	thalamus	94158	93285
53	CJ1301_slide91unsharp_thalamus_R_1	R	thalamus	152284	96060
54	CJ1301_slide91unsharp_thalamus_R_2	R	thalamus	135308	95043
55	CJ1301_slide91unsharp_vtcl_L	L	ventricle	92600	67573
56	CJ1301_slide91unsharp_vtcl_med	C	ventricle	1158889	76022
57	CJ1301_slide91unsharp_WM_L	L	white matter	75488	58650
58	CJ1301_slide91unsharp_WM_R	R	white matter	154657	60546

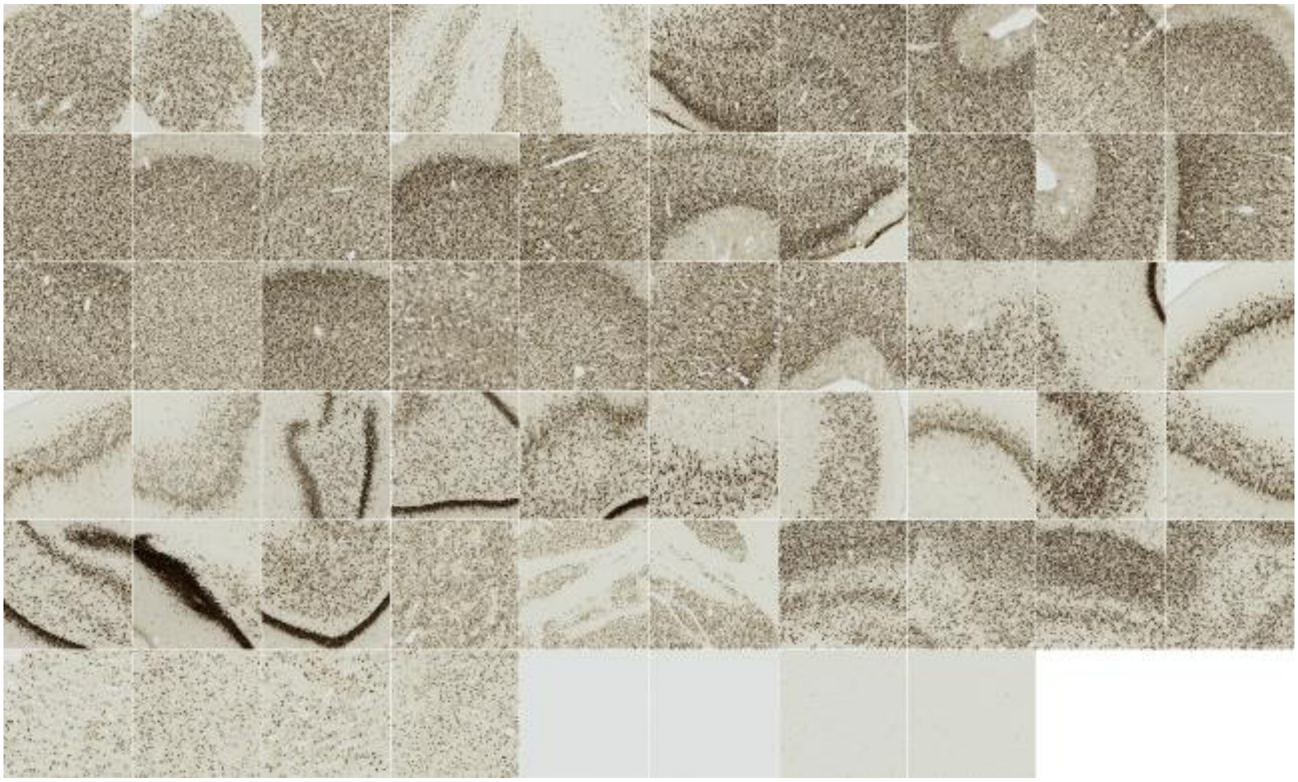

Supplementary Figure 5. *Individualization dataset* images for the individualization of neurons. From left to right, from top to bottom: images are numbered from N° 1 to N° 58. Thereinto, the images of N° 3, 24, 25 and 46 were so fuzzy that expert couldn't distinguish neurons.

Note:

- The images of N° 1-2, 4-23, 26-45, 47-54 were used to compare the number of neurons estimated automatically and manually.
- The images of N° 1, 4, 7, 12, 33-34, 44, 47, 51 were used to study the location of the centroids and the contours of the neurons.

4 Dataset for the quantitative stereological counting – *stereology dataset*

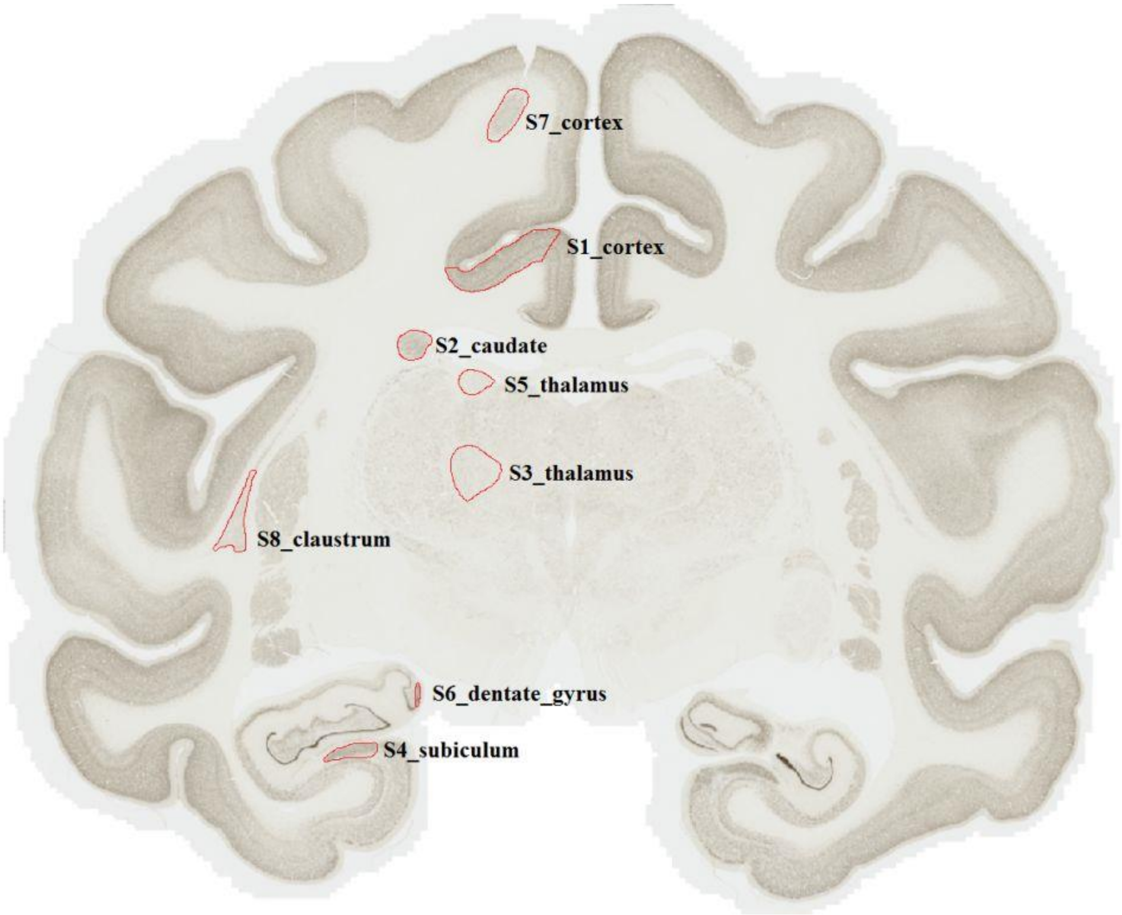

Supplementary Figure 6. Image of brain section N° 91. The regions delineated in red are the regions of interest selected to carry out the quantitative stereological counting which was used as the ground truth to validate the proposed method of individualization. S1 - 8 correspond to the number of the ROIs.

5 Stereological counting

Two experts (biologists b1 and b2) who master stereology techniques estimated the number of neurons on the *stereology dataset* (Supplementary Figure 6) dedicated to stereological counting. Each biologist counted the neurons twice independently on the same anatomical region directly under a microscope using Mercator (Explora Nova, Supplementary Figure 7). In each anatomical region, the biologists first traced the contours of the region to be studied (both expert used the same delineated contours), and then performed random systematic sampling using a number of optical dissectors with the optimal parameters determined in order to obtain an unbiased estimation (the optical dissector size, the distance between two optical dissectors are displayed in "Sampling" column in Supplementary Table 4). The sampling fraction ranged from 1/100 to 1/4 in this study. In each optical dissector (Supplementary Figure 7B), there was a green border (the acceptance line) and a red border (the rejection line) that gave the rules for taking or not into account the neurons that intersect them. The biologists then pointed out the centers of neurons that were located inside the optical dissectors or touching the green borders. Neurons touching the red borders were not counted. Working directly under microscope, it is possible for biologists to modify the focus according to the section depth which facilitates the identification of overlapping or cut neurons. In order to be able to compare with image processing approach which deal with flatten images (single focus setting), we adapted this technique and we did not take into account dead zones.

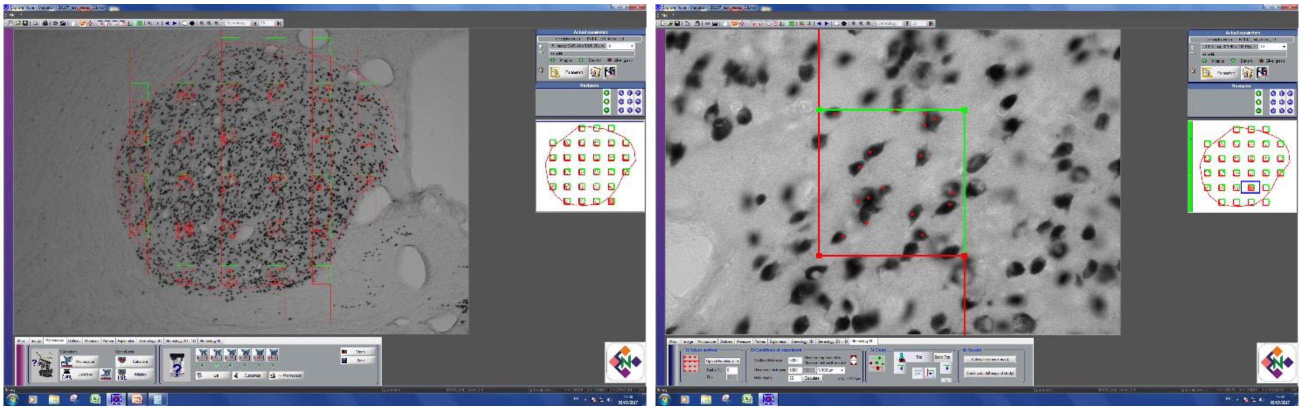

A B

Supplementary Figure 7. (**A**) Mercator software. The biologist first delineated the region of interest – the red outline corresponding to the caudate region. With the optimal parameters, a sampling of optical dissectors was performed and the neurons were counted by the biologists. (**B**) Illustration of the counting of neurons in an optical dissector. Neurons are counted if they are entirely inside the optical dissector or if they intersect the acceptance lines (green) without touching the rejection lines (red). The small red square on neuron are centers pointed out by biologists.

Once the neuron counting achieved on this sampling, the software Mercator have estimated the total number of neurons in this region according to the equation calculated as:

$$N_{total} = \frac{1}{ssf} \times \frac{1}{asf} \times \frac{1}{hsf} \times \sum Q^{-} = \frac{1}{asf} \times \sum Q^{-}$$

where *ssf* is the section sampling fraction, *asf* is the area sampling fraction, *hsf* is the height of the dissector sample relative to the section thickness, N_{total} is the total number of neurons counted in the optical dissectors. In this study, *ssf*=1, *hsf*=1.

Supplementary Table 4. Information of the second sampling about stereological counting, concerning sampling fraction for each anatomical region (the optical dissector (OD) size (µm × µm) / the square of the distance between two optical dissectors (µm)), the number (Nb) of counted optical dissector, the number of counted neurons in all optical dissectors, the total area of all optical dissectors, the region area and the number of neurons evaluated in each anatomical region. In this study, *asf*=Counted area / Region area. Nb of evaluated neurons = Nb of counted neurons / (Counted area / Region area).

Regions	Expert	Sampling	Nb of counted OD	Nb of counted neurons	Counted area (µm^2)	Region area (µm^2)	Nb of evaluated neurons
S1_cortex	b1	50×50/400×400	28	226	70000	5892227	19023
	b2	50×50/400×400	28	211	70000		17761
S2_caudate	b1	50×50/100×100	63	263	157500	1558283	2602
	b2	50×50/100×100	68	266	170000		2438
S3_thalamus	b1	50×50/200×200	62	88	155000	4239108	2407
	b2	50×50/200×200	69	90	172500		2212
S4_subiculum	b1	50×50/200×200	15	107	37500	1235170	3524
	b2	50×50/200×200	18	130	45000		3568
S5_thalamus	b1	50×50/100×100	55	64	137500	1327570	618
	b2	50×50/100×100	57	49	142500		456
S6_dentate_gyrus	b1	20×20/40×40	29	88	11600	126718	961
	b2	20×20/40×40	30	98	12000		1035
S7_corte×	b1	50×50/400×400	11	45	27500	2332417	3817
	b2	50×50/400×400	11	57	27500		4834
S8_claustrum	b1	50×50/200×200	39	172	97500	2309251	4074
	b2	50×50/200×200	34	127	85000		3450

6 Statistics calculated for average neuron radius in different anatomical regions

Supplementary Table 5. Six summary statistics calculated for average neuron radius in different anatomical region. The unit is micrometer.

Anatomical regions	min	1st Qu.	median	mean	3rd Qu.	max
Caudate	1.26	4.02	5.05	4.91	5.79	10.36
Claustrum	1.28	2.99	3.69	3.80	4.52	9.30
Cortex	1.34	3.74	4.64	4.77	5.69	17.80
Hippocampus	1.02	3.96	4.91	5.08	6.02	20.38
Putamen	1.44	3.31	3.98	4.033	4.73	9.41
Subiculum	1.37	4.24	5.16	5.08	5.94	11.71
Thalamus	1.29	3.34	4.21	4.49	5.48	11.64
